# Combinatorial Control of Light Induced Chromatin Remodeling and Gene Activation in *Neurospora*

**DOI:** 10.1371/journal.pgen.1005105

**Published:** 2015-03-30

**Authors:** Cigdem Sancar, Nati Ha, Rüstem Yilmaz, Rafael Tesorero, Tamas Fisher, Michael Brunner, Gencer Sancar

**Affiliations:** 1 Biochemistry Center, University of Heidelberg, Heidelberg, Germany; 2 Institute of Human Genetics, University of Ulm, Ulm, Germany; Charité—Universitätsmedizin Berlin, GERMANY

## Abstract

Light is an important environmental cue that affects physiology and development of *Neurospora crassa*. The light-sensing transcription factor (TF) WCC, which consists of the GATA-family TFs WC1 and WC2, is required for light-dependent transcription. SUB1, another GATA-family TF, is not a photoreceptor but has also been implicated in light-inducible gene expression. To assess regulation and organization of the network of light-inducible genes, we analyzed the roles of WCC and SUB1 in light-induced transcription and nucleosome remodeling. We show that SUB1 co-regulates a fraction of light-inducible genes together with the WCC. WCC induces nucleosome eviction at its binding sites. Chromatin remodeling is facilitated by SUB1 but SUB1 cannot activate light-inducible genes in the absence of WCC. We identified FF7, a TF with a putative O-acetyl transferase domain, as an interaction partner of SUB1 and show their cooperation in regulation of a fraction of light-inducible and a much larger number of non light-inducible genes. Our data suggest that WCC acts as a general switch for light-induced chromatin remodeling and gene expression. SUB1 and FF7 synergistically determine the extent of light-induction of target genes in common with WCC but have in addition a role in transcription regulation beyond light-induced gene expression.

## Author Summary

In this study we have investigated the roles of the *Neurospora* transcription factors (TFs) WCC and SUB1 in light-activation of transcription. In principle TFs could exert identical functions for transcriptional activation and the extent of transcription will be determined by the sum of activity of the TFs. In this case however, we found that the activity of the main blue-light photoreceptor WCC is essential for the activation of light-inducible genes. SUB1 cooperates synergistically with the WCC to enhance expression of a subset of genes controlled directly by the light-activated WCC but cannot activate its light-inducible target genes in the absence of WCC. WCC evicts nucleosomes at its binding sites. This process is supported by SUB1 at a subset of common target genes. Light-dependent nucleosome loss generally correlates with but is not dependent on induction of transcription. Light-induced nucleosome eviction by the WCC/SUB1 could sensitize promoters for activation via endogenous and exogenous cues other than light, which may modulate the plasticity of the light-responsive transcriptome.

## Introduction

Organisms synchronize their behavior and physiology with the geophysical day-night cycle by acute signal transduction of rhythmically reoccurring cues and via anticipatory processes controlled by circadian clocks. Circadian clocks are biological timing systems that operate from the cellular to the organismal level. They are crucially dependent on interconnected transcriptional and posttranscriptional feedback loops that are intimately connected with metabolism [1,2,3,4]. Although dispensable for clock function per se, light is generally a strong cue for the synchronization of endogenous circadian oscillations with the environmental day-night cycle [5,6,7]. Light also induces acute transcriptional responses, particularly in photosynthetic organisms and in fungi [8,9].

White Collar 1 (WC1) is the major blue-light photoreceptor of *Neurospora crassa*. WC1 and its partner WC2 are GATA-family DNA binding proteins that assemble into the hetero-dimeric transcription factor (TF) White Collar Complex (WCC) [10,11]. WCC is essential for light-induced gene expression. It regulates carotenoid biosynthesis, asexual spore formation (conidiation) and sexual reproduction [12]. WC1 contains a flavin-binding light-oxygen-voltage (LOV) blue-light photoreceptor domain [13,14,15]. Light exposure of such LOV domains induces a covalent flavin-cysteinyl photo-adduct and formation of LOV domain dimmers [16,17,18]. Light-activation of WCC results in dynamic homo-dimerization of WCC protomers, which then bind to specific light-responsive DNA elements (LREs) to activate transcription of target genes [15,19,20,21,22]. WCC is also the core TF of the circadian clock of *Neurospora*. It supports self-sustained circadian gene expression rhythms in the dark and synchronizes the circadian oscillator with rhythmic exogenous light cues [2,23,24,25,26]. The dark form of WCC supports a low amplitude circadian nucleosome occupancy rhythm at the so-called clock-box in the *frq* promoter [27,28] while light-activated WCC supports nucleosome remodeling at the light-responsive element (LRE) close to the transcriptional start site [29]. Light-activated WCC enhances transcription of the *sub1* gene, which encodes a GATA-family TF [19,30]. It has been proposed that light-induced accumulation of SUB1 drives in a hierarchical fashion expression of a subset of so-called late light-responsive genes on a second tier [30]. However, substantial levels of *sub1* are already expressed in the dark [19,30,31] and SUB1 target genes are still light-inducible to a lower extent in the absence of SUB1 [30] suggesting a more complex regulation of SUB1-dependent light-inducible genes.

We show here that SUB1 cannot activate transcription of light-inducible genes in the absence of WCC. Rather, such genes are activated by WCC and SUB1 supports the activity of WCC. Light-activation of WCC is associated with nucleosome eviction at its binding sites and target promoters. Efficient remodeling of some nucleosomes is dependent on SUB1. SUB1 interacts with the TF Female Fertility 7 (FF7). SUB1 and FF7 contribute to transcription of a subset of light-inducible genes but are also required for efficient expression of a large number of non light-inducible genes.

## Results

### SUB1 is required for efficient light induction of genes

We analyzed expression of SUB1 in the dark and light to confirm that *sub1* is a direct target of WCC as shown previously [19,30]. When *Neurospora* was grown in constant darkness SUB1 was rhythmically expressed (S1A Fig.). Basal expression levels of SUB1 were essentially independent of WCC but upon light exposure expression of SUB1 was rapidly induced in a WCC-dependent manner (S1B Fig.). The data demonstrate that *sub1* is a light-induced and clock-controlled gene.

**Fig 1:**
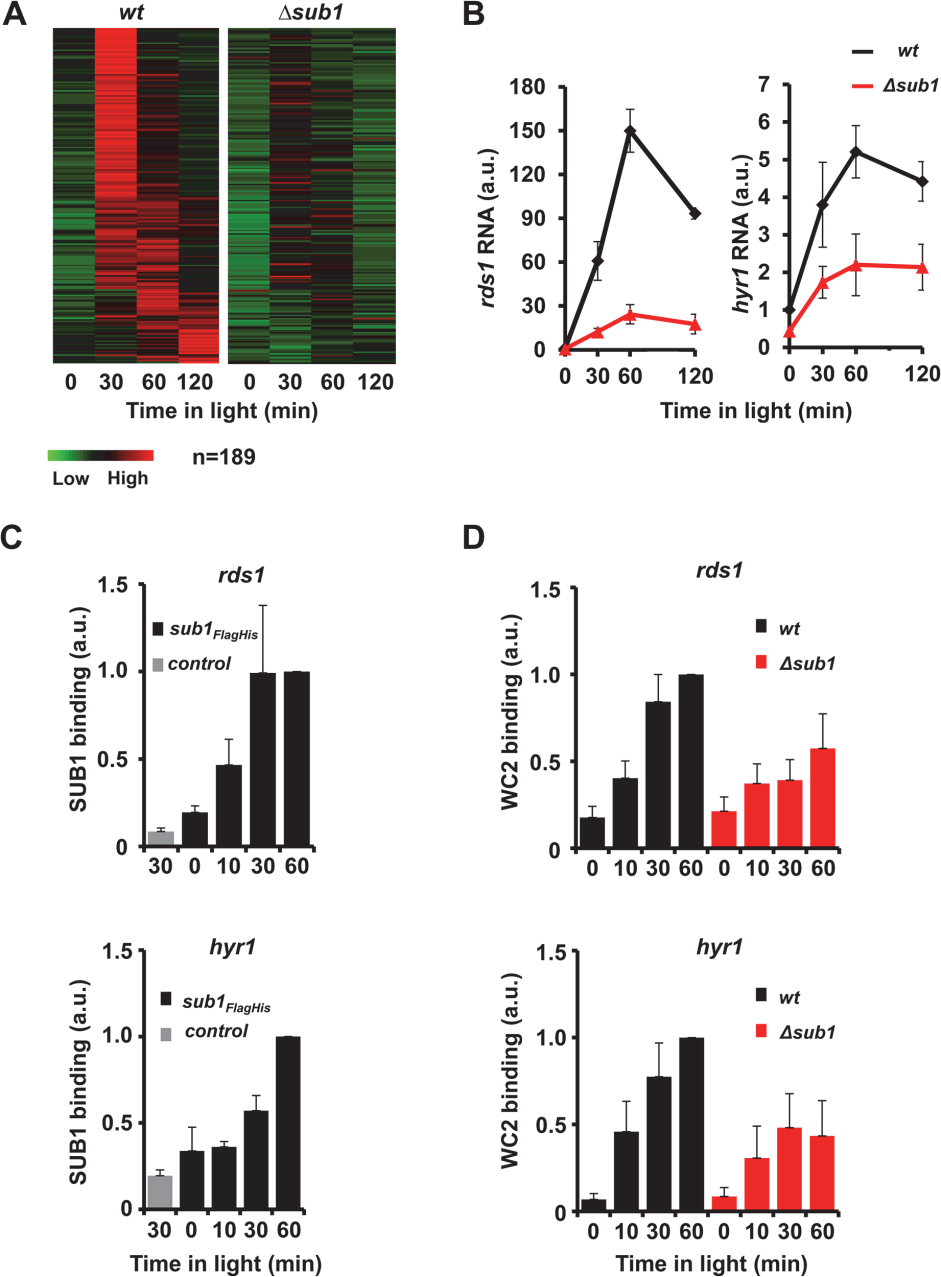
SUB1 is required for efficient recruitment of WCC and light-induction of a subset of genes. **A**. Heat-maps showing the 189 light-inducible genes with significantly lower light-induced RNA levels in Δ*sub1* compared to *wt*. **B**. Quantification of light-induced RNA levels of *rds1* and *hyr1* by RT-PCR (± SEM, n = 4). 28s rRNA was used for normalization. *wt* RNA levels in dark were normalized to 1. **C**. ChIP-PCR analysis of SUB1 showing binding of SUB1 to the *rds1* (upper panel) and *hyr1* promoters (lower panel) determined by two-step FLAG-HIS ChIP. *wt*_*9718*_ was used as a control. 28s rDNA was used for normalization. SUB1 binding at t = 60 min was set to 1 (± SEM, n = 3). **D**. ChIP-PCR analysis of WC2 showing light-induced binding of the WCC to the *rds1* (upper panel) and *hyr1* promoters (lower panel) in *wt* and Δ*sub1* strains. 28s rDNA was used for normalization. WC2 ChIP at t = 60 min in *wt* was set to 1 (± SEM, n = 4).

To investigate a possible crosstalk of the GATA-family TFs SUB1 and WCC we analyzed by RNA-seq the transcriptomes of *wt* and Δ*sub1* strains grown in the dark and after light-exposure. We identified 519 light-inducible genes in *wt* (S1C Fig., S1 Table). 319 of these genes were also identified recently in a similar analysis [31]. More than 80% of the light-inducible genes (417) identified in our study responded rapidly to the light cue (>2x induction within 30 min), strongly suggesting that these are immediate early genes directly controlled by WCC. About 50% of the transcripts accumulated to maximal levels after 30 min and the RNA levels decreased subsequently (early genes). The remainder of the transcripts reached peak expression levels somewhat later (late genes). Light-induction of a substantial subset of genes (189) was severely impaired in Δ*sub1* (Fig. 1A). Both early and late accumulating light-induced transcripts were affected in Δ*sub1*. The temporal transcription dynamics of SUB1-affected genes corresponds to the transient activity profile of light-activated WCC suggesting that SUB1 may cooperate with WCC rather than acting independently and downstream of WCC. In addition, deletion of *sub1* affected expression of 593 genes that were not induced by light, indicating a major role of SUB1 beyond regulation of light-induced transcription (S1D Fig.).

We then analyzed by qPCR the SUB1-dependent regulation of the light-inducible genes *rds1* and *hyr1*, which harbor a WCC binding site in their promoters [19]. Expression of both genes was rapidly induced by light and the light-induction was attenuated in a *Δsub1* strain (Fig. 1B). In contrast, light-induction of the WCC-dependent *vvd* gene was not significantly affected by SUB1 (S1E Fig.). To assess the SUB1-dependence of light-induced transcription initiation at the *rds1* and *hyr1* promoters we analyzed the recruitment kinetics of RNA polymerase II (RNAPII) in *wt* and *Δsub1* strains. SUB1-dependent recruitment of Ser5 phosphorylated RNAPII, which is indicative of transcription initiation, was already detected 5 min after light-induction (S1F Fig.), i.e. prior to the light-induced accumulation of newly synthesized SUB1 (S1G Fig.). The data indicate that the previously synthesized, old SUB1 cooperates with light-activated WCC to activate transcription at the *rds1* and *hyr1* promoters. Moreover, we analyzed recently published ChIP-seq data of recruitment kinetics of RNAPII in response to a single 1 min light-pulse [32]. The data revealed that 40% of the SUB1-dependent light-inducible genes and 43% of the SUB1-independent genes showed a rapid (5–10 min) and transient increase in RNAPII occupancy (S1H Fig), suggesting that these light-inducible target genes of SUB1 are directly activated by WCC. The rest of SUB1-dependent (60%) and independent (57%) light-inducible genes did not show significant RNAPII recruitment under these conditions (1 min light-pulse), suggesting that longer periods of light exposure are required for their maximal activation.

### SUB1 binding to promoters of *hyr1* and *rds1* enhances WCC recruitment

Since SUB1 and WCC contain GATA-family DNA-binding domains we asked if these TFs bind to the same sites in the *hyr1* and *rds1* promoters. We therefore constructed by gene replacement a strain expressing a FLAG-HIS tagged version of SUB1 and performed tandem chromatin immunoprecipitation (ChIP) [33]. In dark-grown mycelia SUB1_FLAG-HIS_ was detected at a low level at the WCC binding sites in the *rds1* and *hyr1* promoters (Fig. 1C). Recruitment of SUB1_FLAG-HIS_ was enhanced after light exposure of mycelia, suggesting that light-activated WCC facilitates binding of SUB1 directly and/or indirectly by supporting light-induced expression of new SUB1.

To assess if SUB1 binding also affects recruitment of WCC we analyzed binding of WCC in *wt* and Δ*sub1* strains. Light-induced binding of WCC to the *rds1* and *hyr1* promoters was attenuated in a Δ*sub1* strain (Fig. 1D), indicating that SUB1 supports recruitment of light-activated WCC to these promoters. Hence, SUB1 and WCC mutually facilitated their recruitment to overlapping or nearby binding sites in the *rds1* and *hyr1* promoters.

### WCC binds preferentially to tandem GATC motifs

**Fig 2:**
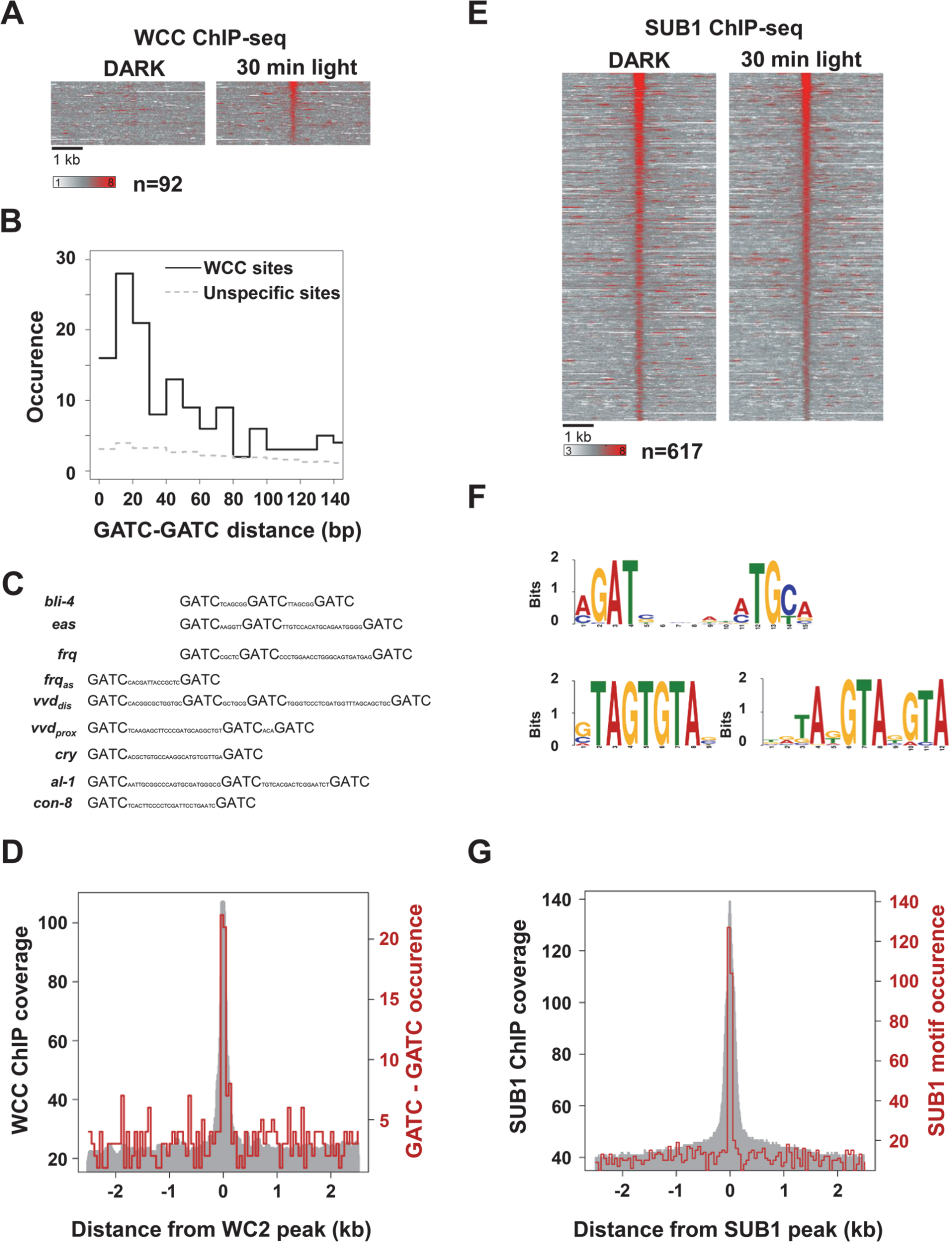
Cistrome analysis of WCC and SUB1. **A**. Heat-map showing the light-induced WCC occupancy at 92 binding sites identified by both, MNase-WC2 ChIP-seq and TAP-WC2 ChIP-seq. 5 kb region covering the binding sites are shown. Left panel: WCC binding in the dark. Right panel: WCC binding 30 min after light-exposure. **B**. Occurrence of tandem GATC motifs with the indicated spacing at WCC binding sites. 300 bp DNA regions covering the peaks of 92 highly confident WCC binding sites were analyzed. The dashed line corresponds to the occurrence of tandem GATC motifs in a set of randomly chosen 300 bp regions. **C**. Potential light response elements (LREs) at WCC binding sites contain multiple GATC motifs. GATC motifs in WCC binding sites of the indicated genes are shown. *frq*_*as*_: *frq* antisense [19]. *vvd*_*prox*_ and *vvd*_*dis*_: proximal and distal WCC binding sites in *vvd* promoter (S2 Table). **D**. Distribution of tandem GATC motifs with < 30 bp spacing at WCC binding sites. The grey area represents the sequence coverage of the WCC ChIP (MNase-WC2 ChIP, 30 min) at the highly confident 92 WCC binding sites. The red line shows the occurrence of tandem GATC motifs. **E**. Heat-map showing the SUB1 occupancy at binding sites. Left panel: SUB1 binding in the dark. Right panel: SUB1 binding 30 min after light-exposure. **F**. SUB1 binding motifs identified by MEME are shown. The major sequence motif shown in the upper panel is found in 171 sites. The GTA-rich motifs shown in the lower left and right panels are present in 82 and 63 sites, respectively. **G**. Distribution of the major SUB1 binding motif (a/cGAT-x6-a/cTGc/t) at SUB1 binding sites. The grey area represents the sequence coverage of the SUB1 ChIP (SUB1 30 min) at 617 SUB1 binding sites. The red line shows the occurrence of the SUB1 binding motif.

We next determined by ChIP-seq the binding sites of WCC on a genome-wide scale. We, in collaboration with others, had previously identified binding sites of light-activated WCC on the basis of a ChIP-seq analysis with rather low sequence coverage [19]. Here, we determined WCC binding sites using two further independent ChIP-seq approaches. In the first approach we fragmented chromatin by sonification and identified 466 light-inducible putative WCC binding sites (S2A Fig., S2 Table) by tandem affinity ChIP of a TAP-tagged WC2 [34]. In the second approach, chromatin was gently fragmented by MNase digestion and we identified 218 putative binding sites of WCC by ChIP with WC2 antibodies (S2B Fig., S2 Table). The combined analyses revealed 582 light-dependent putative WCC binding sites with 92 highly confident binding sites that were identified by both approaches (Fig. 2A, S2 Table).

Analysis of binding motifs by MEME [35] revealed a GATC-containing consensus motif (S2C Fig.), similar to previously identified WCC binding motifs [10,15,19]. Further analysis revealed that WCC sites were enriched in tandem GATC motifs with a preferential pairwise spacing of 10–30 bp (Fig. 2B, C and D). This arrangement of GATC motifs may reflect that light-activated WCC is a dimer of two WC1/WC2 protomers [21] and thus can potentially bind up to four GATC motifs.

The 92 highly confident WCC binding sites were associated with 91 genes and 51 of these genes were light-inducible. This enrichment of light-inducible genes suggests that these binding sites are functionally relevant. 41 WCC binding sites contain a consensus tandem GATC motif. Binding of WCC to 30 of these sites correlated with light-inducible expression of the associated genes. However, 11 sites with a consensus tandem GATC motif were not associated with light-inducible expression of neighboring genes under the conditions analyzed here (S5 Table).

Although the vast majority of light-inducible genes responded rapidly to light-cues (see above) we have not detected highly confident WCC binding sites (n = 92) in all light-inducible genes (n = 519), raising the question of whether they are directly controlled by the WCC. A visual inspection of the WC2 ChIP-seq coverage at promoters of light-inducible genes without significant binding sites revealed putative light-dependent WCC sites that were not detected by our peak-calling algorithm (S2D Fig.). A subsequent ChIP-PCR analysis revealed a light-dependent enrichment of WCC at such regions (S2D Fig.) indicating that they are true WCC binding sites and suggesting that the associated light-inducible genes are directly controlled by the WCC. Hence, not all of the WCC binding sites were detected by our ChIP-seq analysis.

### SUB1 binding to the majority of sites is light-independent

In order to assess the possible effect of light on SUB1 binding on a genome-wide scale we performed tandem ChIP-seq [33] of SUB1_FLAG-HIS_ using dark grown and light-exposed mycelial cultures. We identified 617 binding sites that were associated with 562 genes (Fig. 2E, S2 Table). 63 genes were light-inducible suggesting a moderate but significant enrichment of SUB1 at light-inducible genes (p < e-07). However, binding of SUB1 to the majority of sites (527) was independent of light. Hence, the elevated expression of SUB1 in light-exposed mycelia does not support increased SUB1 binding on a genome-wide scale. Recruitment of SUB1 to 50 sites (associated with 50 genes) was, however, enhanced by light (S2E Fig.) and 27 of the associated genes were light-inducible. Interestingly, 23 of these 27 genes harbor overlapping (S2F Fig.) or close by WCC binding sites. The data indicate that light-induced SUB1 binding occurs mainly at light-inducible WCC target genes.

Analysis of SUB1 binding sites by MEME revealed the bipartite motif a/cGATc/g-x_6_-a/cTGc/t (Fig. 2F, upper panel). This motif was highly enriched (223 / 617) and located in the center of the SUB1 binding sites (Fig. 2G). The MEME analysis revealed in addition two GTA-rich motifs, which were present in 82 and 63 sites, respectively (Fig. 2F, lower panels).

In order to assess the difference between SUB1-dependent and independent light-activated genes we next analyzed recruitment of SUB1 and WCC to light-inducible genes. Sequence coverage of the SUB1 ChIP was higher at promoters of SUB1-dependent light-inducible genes while WCC occupancy was higher at promoters of SUB1-independent light-inducible genes (S2G and S2I Fig.). The data suggest that SUB1 may preferentially support light-induced transcription at promoters with lower affinity for WCC.

### SUB1 supports WCC-dependent light-induced nucleosome eviction

To assess whether WCC and/or SUB1 support light-induced chromatin remodeling we performed nucleosome mapping of dark-grown and light-exposed *wt*, Δ*sub1* and Δ*wc2* strains by MNase digestion of DNA followed by paired-end sequencing. Two independent replicates were analyzed. The average length of protected fragments was about 140–150 bp and fragments longer than 100 bp were considered nucleosomal DNA, while protected smaller fragments were considered footprints of TFs or other DNA-binding proteins.

**Fig 3:**
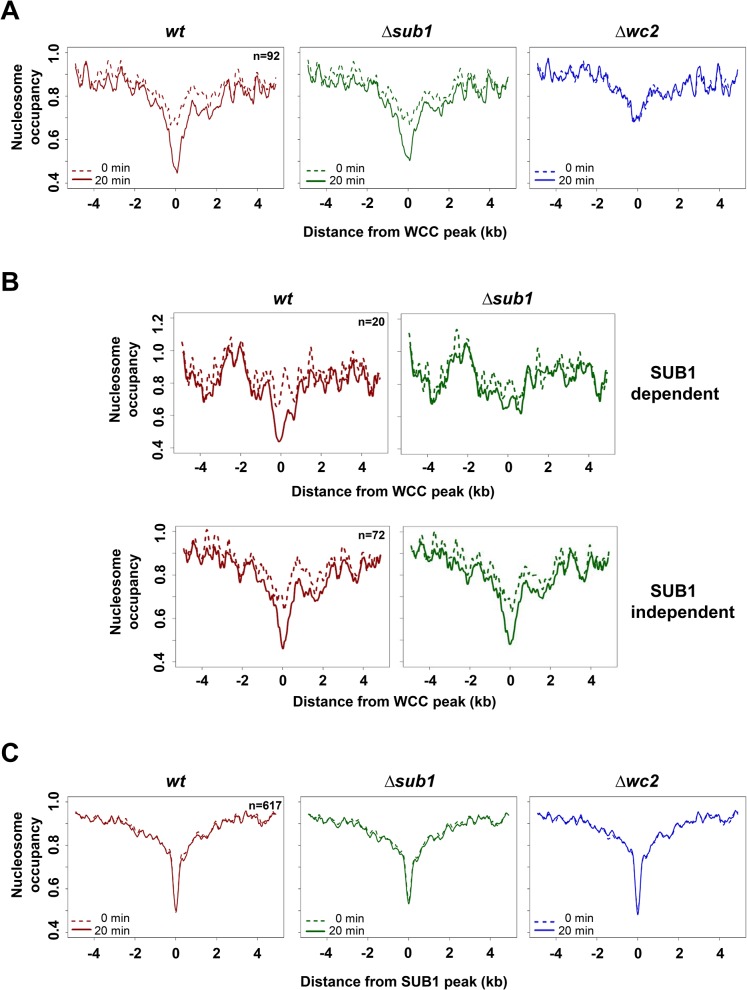
Light promotes WCC dependent nucleosome eviction at WCC binding sites. **A**. Nucleosome occupancy at binding sites of WCC (n = 92) and in *wt* (red), Δ*sub1* (green) and Δ*wc2* (blue) strains in dark (dotted lines) and 20 min after light-exposure (solid lines). **B**. Nucleosome occupancy at binding sites of WCC for SUB1-dependent (upper panel) and SUB1-independent (lower panel) light induced nucleosome loss. *wt* (red) and Δ*sub1* (green) strains in dark (dotted lines) and 20 min after light-exposure (solid lines) are shown. **C**. Nucleosome occupancy at binding sites of SUB1 (n = 617) in *wt* (red), Δ*sub1* (green) and Δ*wc2* (blue) strains in dark (dotted lines) and 20 min after light-exposure (solid lines).

The nucleosome occupancy profiles at WCC binding sites were bimodal. A rather broad region (about ± 2kb) with moderately reduced nucleosome occupancy likely reflects that WCC binding sites are enriched in promoters, which are generally rather nucleosome free, while the confined nucleosome-free region in the center may reflect the actual binding site of WCC (Fig. 3A, dashed lines). In the dark nucleosome occupancy at WCC sites was similar in all the stains (S3A Fig., left panel). Upon light-exposure, the nucleosome occupancy decreased in *wt* and Δ*sub1* but not in Δ*wc2*, demonstrating that the activated WCC reduces nucleosome occupancy at its binding sites (Fig. 3A, solid lines). Maximal depletion of nucleosomes was observed in *wt*, i.e. when SUB1 and light-activated WCC were present (Figs. 3A and S3A, right panel). Analysis of an independent nucleosome analysis supported these results (S3B Fig.). The light-induced nucleosome loss at 20 highly confident WCC binding sites was impaired in Δ*sub1* while nucleosome eviction was independent of SUB1 at the remaining 72 WCC sites (Fig. 3B).

Nucleosome occupancy at SUB1 binding sites was rather low in *wt*, Δ*wc2* and even in Δ*sub1* and essentially independent of light (Figs. 3C, S3A). The data suggest that SUB1 binding sites are either intrinsically free of nucleosomes or that other chromatin remodelers keep these sites open.

To obtain potential footprints of the WCC and SUB1, we analyzed MNase-resistant DNA fragments that were shorter than typical fragments protected by nucleosomes. Protected DNA fragments < 100 bp accumulated in light-dependent fashion at WCC binding sites (S3C Fig., left panel), suggesting that they correspond to a footprint of the light-activated WCC. In contrast, a potential footprint of SUB1 was not affected by light (S3C Fig., right panel).

Together these observations indicate that binding of the light-activated WCC triggers depletion of nucleosomes from its binding sites. SUB1 contributes to the light-induced nucleosome removal at WCC binding sites. Binding sites of SUB1 are also rather devoid of nucleosomes, even in the absence of SUB1, and the nucleosome occupancy of SUB1 sites was independent of light and WCC.

**Fig 4:**
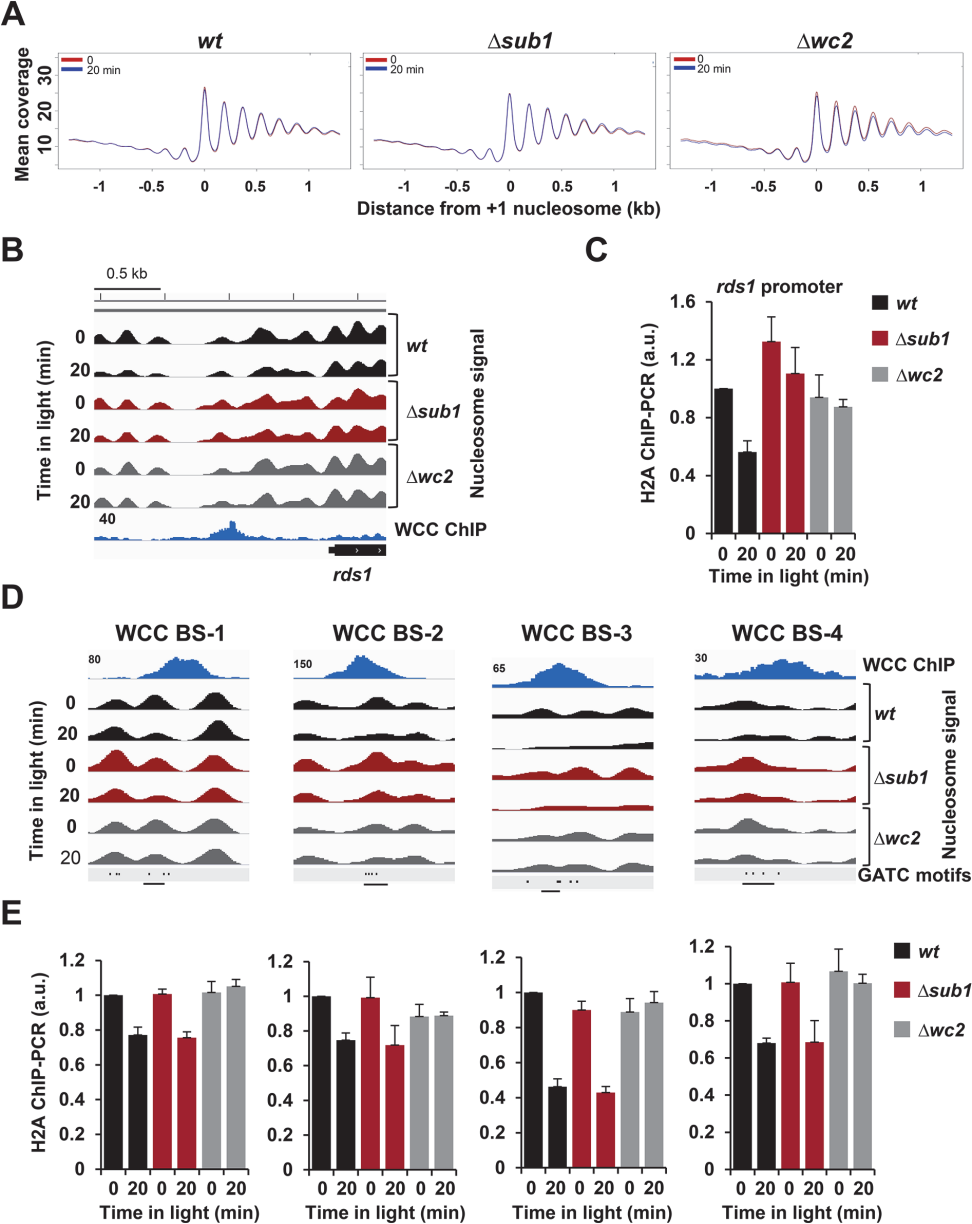
Light- and WCC-dependent nucleosome eviction is transcription independent. **A**. Line graphs showing the averaged nucleosome occupancy in transcribed genes and promoters of all annotated *Neurospora* genes (n = 9733) in *wt*, Δ*sub1* and Δ*wc2* strains in dark (red) and 20 min (blue) after light-exposure of cultures. The center of the +1 nucleosome (nucleosome overlapping the annotated transcription start site) was used for alignment of sequence coverage of MNase-resistant fragments >100bp. **B**. Wig file showing the nucleosome position and occupancy at the *rds1* promoter in *wt*, Δ*sub1* and Δ*wc2* strains in the dark and after light-exposure. MNase-WC2 ChIP-seq (blue) is shown below the nucleosome signals. Numbers on the ChIP-seq panels show the maximum read coverage shown in the wig file. **C**. ChIP-PCR analysis showing H2A occupancy in the dark and 20 min after light exposure at the binding sites of WCC and SUB1 in the *rds1* promoter. ChIP was performed by immunoprecipitation with H2A antibody (± SEM, n = 4). a*ctin* gene was used for normalization. *wt* dark level was set to 1. **D**. Transcription-independent light-induced nucleosome eviction at WCC binding sites (BS). Four examples (wig files) of nucleosome position and occupancy at WCC BS in *wt*, Δ*sub1* and Δ*wc2* strains are shown. WCC binding (TAP-WC2 ChIP-seq) is shown above the nucleosome signals. The positions of GATC motifs are shown in the lower panels. Numbers on the ChIP-seq panels indicate the maximum nucleosome coverage shown in the Wig file. Regions used for ChIP-PCR analysis are indicated by black lines. **E**. ChIP-PCR analysis showing H2A occupancy in the regions shown in Fig. 4D. Occupancy of H2A was determined by immunoprecipitation with H2A antibody (± SEM, n = 4). a*ctin* gene was used for normalization. w*t* dark level was set to 1.

To identify on a genome-wide level light-induced nucleosome remodeling in promoters and genes, we aligned the +1 nucleosomes of all annotated transcription start sites (TSSs). In transcribed regions the nucleosomes were regularly spaced by 176 bp and nucleosome occupancy was rather high (Fig. 4A). In contrast, nucleosome spacing was irregular and occupancy was lower in promoters, similar to corresponding observations in other species [36,37,38]. Light, WCC and SUB1 did not affect nucleosome occupancy of genes and promoters on a genome-wide scale (Fig. 4A). However, light triggered nucleosome remodeling at the promoter of the SUB1-dependent *rds1* gene. A light-induced loss of nucleosomes was detected at the overlapping WCC and SUB1 binding sites (Fig. 4B and C). The light-induced nucleosome loss was attenuated in Δ*sub1* and absent in a Δ*wc2* strain, indicating that removal was strictly dependent on the activated WCC and supported by SUB1. A light-induced loss a nucleosome was also observed at the WCC binding site of the *hyr1* promoter (S4A Fig., left panel), which was, however, not dependent of SUB1.

Light triggered a substantial loss and repositioning of nucleosomes at the *vvd* promoter and gene (S4A Fig., right panel). The light-induced nucleosome dynamics were similar in *wt* and Δ*sub1* but absent in Δ*wc2*, indicating that chromatin remodeling of the *vvd* gene by the light-activated WCC was independent of SUB1. The pronounced depletion of nucleosomes in the transcribed region of *vvd* is likely due to the synchronous activation of the rather strong *vvd* promoter in the entire ensemble of nuclei. Similar losses of nucleosomes were observed in the transcribed region of other highly expressed light-inducible genes such as *al-1*, *cry* and *con-10* but was less pronounced or not detectable in less active genes such as *hyr1*, *ncu00309* and *frq* (S1 Table).

With the exception of highly transcribed genes the light-induced eviction of nucleosomes was generally confined to one or two nucleosomes overlapping the WCC binding sites. We observed eviction of individual nucleosomes at several high affinity binding sites of the WCC that were not associated with transcription initiation of a neighboring or close-by gene (Figs. 4D and S4B). We confirmed the light- and WCC- dependent nucleosome eviction at these sites by independent histone H2A ChIP-PCR (Fig. 4E). These observations suggest that the light-activated WCC supports eviction of nucleosomes at its binding sites independent of transcription.

### SUB1 requires light-activated WCC to stimulate expression of light-inducible genes

**Fig 5:**
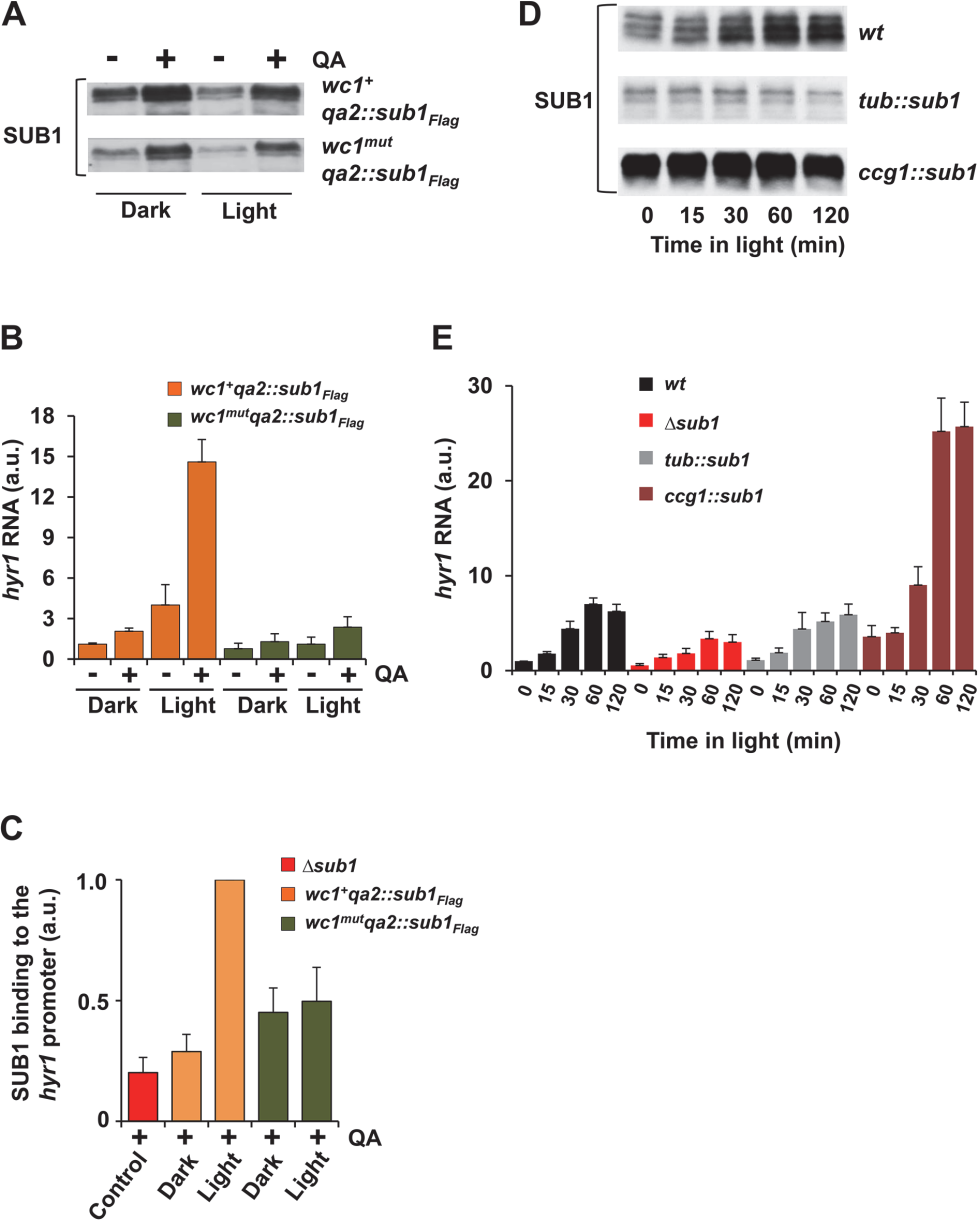
SUB1 requires light-activated WCC to induce *hyr1* gene expression. **A**. Western blot showing SUB1 expression levels of *qa* promoter driven FLAG-tagged *sub1* in *wc1* ^*+*^ and *wc1* ^*mut*^ strains before and 4 h after QA-induction of cultures grown for 24 h in the dark or in constant light. **B**. Quantification of *hyr1* RNA levels by RT-PCR (± SEM, n = 4) under the conditions described in (A). *tubulin* RNA was used for normalization. The *hyr1* RNA level in *wc1* ^*+*^ *qa2*::*sub1*_*FLAG*_ in the dark (-QA) was set to 1. **C**. ChIP-PCR analysis of FLAG-SUB1 showing binding of SUB1 to the *hyr1* promoter in *wc1* ^*+*^ *qa2*::*sub1*_*FLAG*_, *wc1* ^*mut*^_*_*_ *qa2*::*sub1*_*FLAG*_ and Δ*sub1* strains grown 24 h in dark or in constant light. QA-induction, when indicated, was carried out for 4 h. Two-step ChIP was performed using FLAG and SUB1 antibodies. 28s rDNA was used for normalization. The ChIP-PCR signal of light grown *wc1* ^*+*^ *qa2*::*sub1*_*FLAG*_ was set to 1 (± SEM, n = 3). **D**. Western blot showing SUB1 levels after light-exposure of *wt*, *tub*::*sub1* and *ccg1*::*sub1* strains. **E**. Quantification by RT-PCR of light-induced accumulation of *hyr1* RNA levels in *wt*, Δ*sub1*, *tub*::*sub1 and ccg1*::*sub1* strains. 28s rRNA was used for normalization. Dark RNA levels of *wt* were normalized to 1 (± SEM, n = 4).

To address whether SUB1 can activate transcription of light-inducible genes without the WCC we expressed in *wc1*-deficient (*wc1* ^*mut*^) [39] and *wc1*-proficient (*wc1* ^*+*^) strains a FLAG-tagged SUB1 under control of the inducible *quinic acid 2* (*qa2*) promoter. SUB1_FLAG_ was expressed at low level in the absence of QA and expression levels were elevated in the presence of QA (Figs. 5A, S5A). Expression levels of *hyr1*, which is a SUB1-affected light-inducible gene, were generally higher in a *wc1* ^*+*^ background than in the corresponding *wc1* ^*mut*^ strains (Fig. 5B). In light, QA-induced SUB1_FLAG_ supported expression of *hyr1* at a high level in the presence of WCC (*wc1* ^*+*^) but not in the absence of WCC (*wc1* ^*mut*^) (Figs. 5B and S5B). Together the data indicate that SUB1 cannot activate expression of *hyr1* independently of the WCC. Maximal expression of *hyr1* requires the presence of SUB1 and light-activated WCC.

To analyze binding of SUB1_FLAG_ to the *hyr1* promoter we performed ChIP-PCR. In a *wc1* ^*+*^ background binding of QA-induced SUB1_FLAG_ was more efficient in light than in dark while binding of SUB1 was independent of light in a *wc1*-deficient strain (Fig. 5C). The data suggest that the light-activated WCC supports recruitment of SUB1 to the *hyr1* promoter. Corresponding results were obtained for the *rds1* promoter (S5C Fig.).

Since the WCC facilitates recruitment of SUB1, expression of SUB1-dependent light-inducible genes could be limited by SUB1 abundance. To test this hypothesis, we generated WCC-proficient strains expressing SUB1 under control of the *tubulin* (*tub*) and the *ccg1* promoter, respectively. Dark-grown mycelial cultures of *wt*, Δ*sub1*, *tub*::*sub1* and *ccg1*::*sub1* were exposed to light and SUB1 levels and the kinetics of *hyr1* expression were measured (Fig. 5D and E). In *wt*, SUB1 was expressed at low level in the dark and accumulated, as expected, to high levels after light exposure. In contrast, SUB1 levels were constitutively low in *tub*::*sub1* and constitutively high in *ccg1*::*sub1* (Fig. 5D). In the dark *hyr1* expression correlated well with the SUB1 levels in the respective strain. In response to light, *hyr1* levels increased with similar kinetics (5–7 fold) in all strains, reaching the highest expression in *ccg1*::*sub1* and the lowest level in Δ*sub1*. Interestingly, *hyr1* levels were similar in *wt* and *tub*::*sub1* despite light-induced accumulation of substantial amounts of SUB1 in *wt* (Fig. 5D upper panel). Hence, the SUB1 that was newly synthesized under control of the WCC did not independently activate *hyr1* on a second hierarchical tier. Corresponding results were obtained when light-induced expression of the SUB1-dependent genes *rds1* and *ncu00309* was analyzed (S5D, S5E Fig.). Over-expression of SUB1 in a WCC-deficient background (S5F Fig.) did neither support elevated expression of the SUB1 target genes (*rds1* and *ncu00309*) in the dark nor in light (S5G and S5H Fig.). Together the data indicate that SUB1 functionally cooperates with the dark-form and the light-activated WCC but cannot activate transcription of light-inducible genes in the absence of WCC. Thus, SUB1 supports the activity of WCC in synergistic manner but is not a bona-fide transcription activator of light-inducible genes.

We noted that light-induction of *rds1* was strongly attenuated by deletion of *sub1* but was not affected by SUB1 overexpression. In contrast, light-induction of *hyr1* was only moderately affected by deletion of *sub1* but was strongly enhanced by SUB1 overexpression, suggesting that the SUB1 level in *wt* was limiting for *hyr1* expression under the conditions analyzed. To detect *hyr1*-type genes that respond only to high levels of SUB1 we analyzed the light-inducible transcriptome of *ccg1*::*sub1* in comparison to a *wt* strain. In *wt* (replicate 2) we identified 657 light inducible genes (S5I Fig., S3 Table). Most of these genes were also identified by the independent analysis (replicate 1) described above (S5J Fig.). Light-induction of 121 genes was significantly enhanced in *ccg1*::*sub1* (S5K and S5L Fig.). Together with the group of genes down-regulated in Δ*sub1* (see Fig. 1) the data indicates that about 40% (264) of the light-inducible genes are co-regulated by SUB1.

### SUB1 interacts and cooperates with FF7 to activate light-inducible and non light-inducible genes

**Fig 6:**
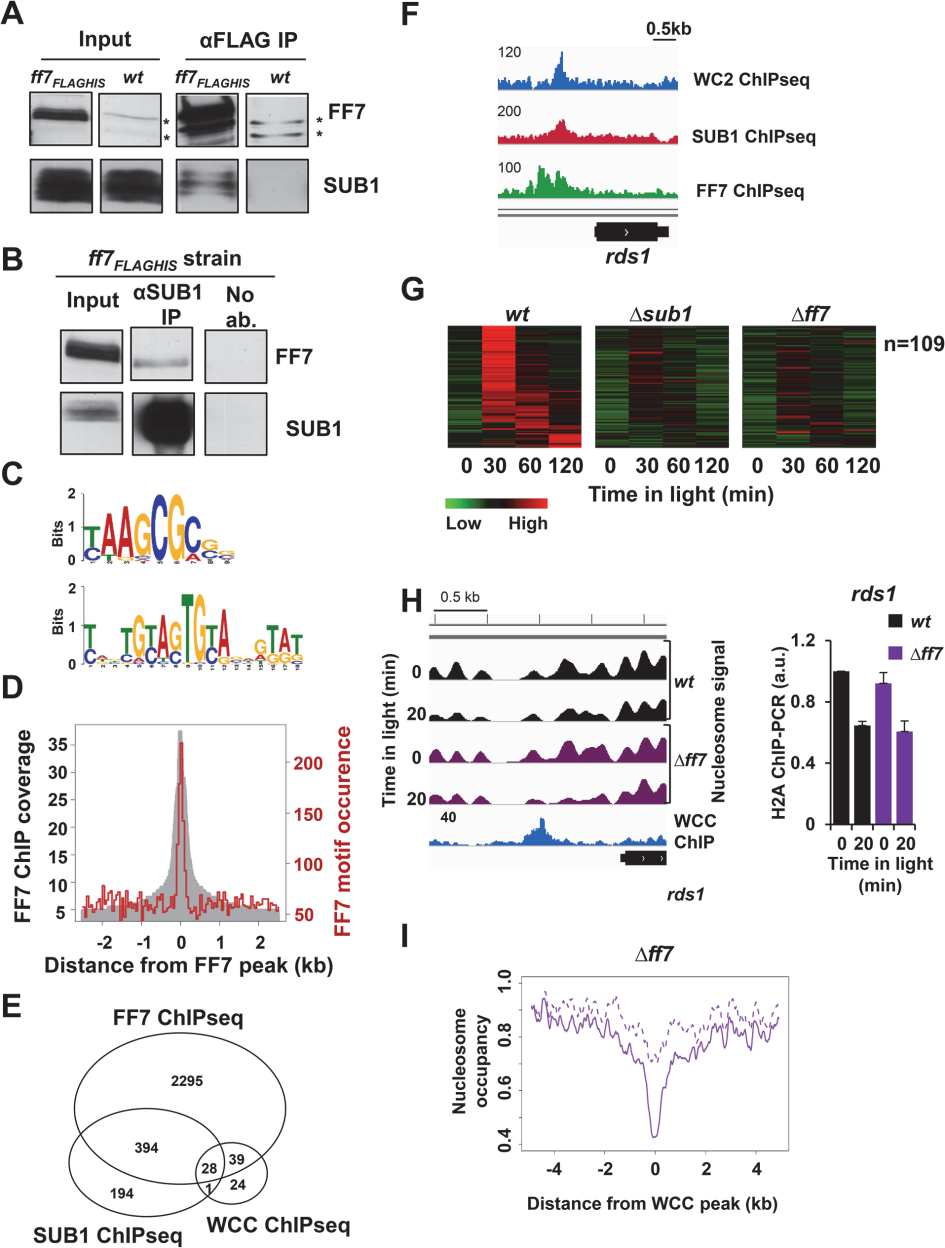
FF7 interacts weakly with SUB1 and co-regulates light-inducible and non light-inducible genes. **A-B**. Western blots showing co-immunoprecipitation (co-IP) of **(A)** SUB1 with FF7_FLAG-HIS_ and **(B)** FF7_FLAG-HIS_ with SUB1. FLAG antibody was used for FF7_FLAG-HIS_ IP and α-SUB1 antibody was used for SUB1 IP. The asterisks (*) indicate cross-reactions of the FLAG antibody. **C**. FF7 binding motifs identified by MEME. The top 200 binding sites identified by FF7 ChIP-seq were used for the motif analysis. The upper motif is found in 117 / 200 binding sites whereas the lower motif is found in 36 / 200 binding sites. **D**. Occurrence of the major FF7 motif at FF7 binding sites. The grey area shows the occupancy of FF7 binding sites determined by ChIP-seq. The red line shows the occurrence of the FF7 binding motif “t/c AAGCG c/a”. **E**. Wig file showing MNase-WC2, SUB1 and FF7 ChIP-seq signals at the *rds1* promoter. Numbers on the ChIP-seq panels correspond the maximum coverage shown in the wig file. **F**. Venn-diagram showing the overlap between SUB1, WC2 and FF7 ChIP-seq signals. **G**. Heat-map showing light-inducible genes with significantly lower RNA levels in Δ*sub1* and in Δ*ff7* strains in comparison to *wt*. **H**. Wig file (left panel) showing the nucleosome position and occupancy at the *rds1* promoter in *wt* and Δ*ff7* strains in the dark and after light-exposure. The MNase-WC2 ChIP-seq (blue) is shown below the nucleosome signals. Numbers on the ChIP-seq panels show the maximum coverage shown in the wig file. ChIP-PCR analysis (right panel) of H2A occupancy at the binding sites of WCC and SUB1 at *rds1* promoter in the dark and 20 min after light-exposure (± SEM, n = 4). a*ctin* DNA was used for normalization. w*t* dark level was set to 1. **I**. Nucleosome occupancy at binding sites of WCC (n = 92) and in Δ*ff7* in dark (dotted lines) and 20 min after light-exposure (solid lines).

To identify interaction partners of SUB1 we performed tandem affinity purification of SUB1_FLAG-HIS_. By subsequent mass-spectrometry we identified Female Fertility-7 (FF7) as a potential interaction partner of SUB1. FF7 has a Gal4-type Zn(2)-Cys(6) binuclear cluster domain and a putative acetyl transferase domain with similarity to maltose acetyl transferase. To confirm the interaction we constructed a strain expressing FLAG-HIS tagged FF7 and performed reciprocal anti-FLAG and anti-SUB1 immunoprecipitations. SUB1 co-immunoprecipitated with FF7_FLAG-HIS_ (Fig. 6A) and vice versa, FF7_FLAG-HIS_ was pulled down with SUB1 antibodies (Fig. 6B). The pull-down efficiency was quite low, suggesting that the interaction is rather unstable. To identify the binding sites of FF7 and to investigate whether SUB1 and FF7 co-localize on the genome, we performed tandem ChIP-seq of FF7_FLAG-HIS_ from light-exposed mycelial cultures. We identified 2756 putative FF7 binding sites that were associated with 2315 genes (S4 Table), suggesting a rather ubiquitous role of FF7. Analysis of FF7 binding sites by MEME revealed a AACCGC motif (Fig. 6C, upper panel) that was highly enriched in the center of the FF7 binding sites (Fig. 6D). A “GTA” rich motif, similar to the one found in the SUB1 ChIP-seq, was also found in ChIPed FF7 sites (Fig. 6C, lower panel). This motif might be a more general element associated with promoters since it was not enriched at the center of the binding sites. To assess the potential relationship of FF7, SUB1 and WCC we analyzed the occupancies of the transcription factors at their own and the respective binding sites of the other TFs (S6A Fig.). FF7 binding was enriched at WCC and at SUB1 sites. Similarly, SUB1 binding was enriched at WCC and at FF7 sites. WCC sites were, possibly due to their low number (n = 92), neither enriched at SUB1 sites (n = 617) nor at FF7 sites (n = 2756). On a genome wide scale, about 70% (422 / 617) of the SUB1 binding sites were also occupied by FF7 indicating a highly significant (p < e-10) co-occurrence of these factors on the DNA (Fig. 6E). Similarly, 72% (68 / 92) of the WCC binding sites overlap with FF7. Interestingly, essentially all SUB1 and WCC overlapping sites (28 / 29) harbor also FF7 binding sites (Fig. 6E). Examples are shown in Figs. 6F and S6B. The 28 overlapping WCC, SUB1 and FF7 binding sites were associated with 25 expressed genes. 10 of these genes were light-inducible and dependent on SUB1 and/or FF7, 5 genes were light-inducible but independent of SUB1/FF7 and 10 genes were not light-inducible (S1 and S6 Tables). Thus, the majority of functional WCC sites with overlapping SUB1 and FF7 sites are also co-regulated by SUB1 and FF7.

To analyze the functional cooperation of FF7 with SUB1 and WCC, we determined by RNA-seq the transcriptome of a Δ*ff7* strain grown in the dark and after light-exposure. RNA levels of 192 of 519 light-inducible genes were reduced in Δ*ff7* (S6C Fig., S1 Table). 109 of these genes show impaired light-induction in Δ*ff7* and in Δ*sub1* (Fig. 6G). We confirmed the impaired light-induction of the *rds1* gene by independent RNA measurements (S6D Fig.). Furthermore, expression of 440 genes was reduced in Δ*ff7* in a light-independent manner (S1 Table) and expression of 278 of these genes was also reduced in Δ*sub1* (S6E Fig.).

Finally, we performed nucleosome mapping of Δ*ff7* after light-induction to assess whether FF7 contributes to light-induced nucleosome eviction at WCC binding sites. The light-induced nucleosome loss at the *rds1* promoter was independent of FF7 despite impaired light-induction of the *rds1* gene in Δ*ff7* (Fig. 6H). Similarly, we did not detect impaired light-induced nucleosome removal at other WCC sites in Δ*ff7* (Fig. 6I) suggesting that FF7 is required for transactivation rather than eviction of nucleosomes. Together the data suggest that WCC, SUB1 and FF7 have distinct functions and cooperate to regulate subsets of genes in a combinatorial fashion.

## Discussion

WC1 and WC2, which constitute the WCC, and SUB1 are GATA-family transcription factors of *Neurospora crassa*. Here we analyzed their roles in regulation of light-induced nucleosome remodeling and gene expression. We identified about 500 light-inducible genes. SUB1 regulates a substantial subset of light-inducible genes (264) in cooperation with the WCC and a larger number of non light-inducible genes. However, SUB1, even when overexpressed, cannot induce transcription of its light-inducible target genes in the absence of WCC. Hence, SUB1 does not independently activate a subset of late light-inducible genes on a second hierarchical tier. The immediate light-induced recruitment of RNAPII, even in response to a 1 min light-pulse, suggests that more than 40% of the light-induced SUB1 target genes are directly activated by the WCC photoreceptor rather than indirectly via light-induced synthesis and accumulation of a TF acting on a second hierarchical tier.

How does SUB1 cooperate with the WCC to regulate light-inducible gene expression? Transcription activation in eukaryotes is based on regulation of DNA accessibility to RNAPII. This is often achieved by cooperation of several transcription regulators and co-regulators facilitating in combinatorial fashion modification and eviction of histones and subsequent recruitment of general transcription machinery and RNAPII [40,41,42,43,44,45]. Conceptually the light-activated WCC and SUB1 could exert identical functions. In this case the TFs would contribute independently and in approximately additive manner to the transcriptional output of common target genes. This is obviously not the case since SUB1 cannot activate its light-inducible target genes in the absence of WCC. Rather, it seems likely that WCC and SUB1 contribute distinct functions to activate common target promoters.

We show that nucleosome occupancy profiles at binding sites of WCC and SUB1 are rather low even in the absence of the cognate TF, suggesting that they are either intrinsically nucleosome-free or that sequence specific machinery keeps these sites open. Additional light-induced eviction of nucleosomes at WCC binding sites is strictly dependent on the WCC. SUB1 contributes to the WCC-dependent light-induced nucleosome loss at a subset of promoters, suggesting that WCC and SUB1 act in combinatorial rather than additive fashion. Light-induced eviction of nucleosomes at WCC binding sites is independent of transcription. The remodeling activity associated with the WCC might be similar to the pioneer-like activity of circadian transcription factor CLOCK/BMAL1 in mice and prepare promoters for activation for other TFs [46]. The light-activated WCC recruits NGF1, a H3K14 acetyl transferase homologous to yeast *GCN5* [47] *and may*, similar to its less active dark form, also recruit the rather ubiquitous ATP-dependent chromatin remodeler SWI/SNF [28] to evict nucleosomes as reported for the promoter of the *frq* [29].

How are the differentially regulated subsets of SUB1-dependent and independent light-inducible genes defined? The highly homologous Zn-fingers of WC1, WC2 and SUB1 bind GATC-related core sequence motifs and flanking sequences seem to distinguish their specificity. Although binding motifs of the dark form of the WCC have not been determined it seems to interact with GATG motifs in the clock box of the *frq* promoter [48] and presumably also with GATC motifs present in the *vvd* LRE [49]. Deletion or mutation of the Zn-fingers of WC1 or WC2 abolishes WCC activity in the dark [50,51]. Hence both Zn-fingers seem to have the capacity to interact with DNA and contribute to WCC activity. When activated by light protomers of WCC dimerize dynamically [21]. The increased ChIP efficiency of light-activated WCC may reflect tighter DNA binding of the WCC dimer, which could potentially interact with up to four GATC-related motifs via the Zn-fingers of two WC1 and two WC2 subunits. Analysis of the 92 highly confident binding sites of light-activated WCC revealed tandem GATC motifs spaced by ≤ 30 bp that are enriched in the center of the binding sites. Strong LREs might thus be determined by number and spacing of GATC motifs. Indeed, highly occupied LREs contain tandem or more GATC motifs (Fig. 2D). Hence, the molecular mechanism underlying light-induced recruitment of WCC seems to be based on a gain in binding avidity. Weak and highly dynamic protein-DNA interactions of WCC protomers with individual GATC related motifs and weak protein-protein interactions between WCC protomers are mutually stabilized at LREs containing properly spaced GATC motifs.

The majority of light-inducible genes were not associated with significant WCC binding sites, i.e. sites detectable by two independent ChIP-seq replicates. The median expression level of light-inducible genes without significant WCC binding site was rather low and many promoters of these genes contain putative low affinity binding sites (S2D Fig.). ChIP-PCR analysis revealed that WCC is in fact recruited in light-dependent fashion to such low affinity sites. Furthermore, promoters of such genes are significantly enriched in tandem GATC repeat motifs. In addition, a large fraction of light-inducible genes without detectable WCC binding sites responded immediately (within a few minutes) even to a short light pulse. These observations suggest that most light-inducible genes are directly activated by the WCC rather than via induction and accumulation of sufficient SUB1 that would then indirectly induce transcription of genes on a second hierarchical tier. However, since the WCC controls expression of several TFs in addition to SUB1 [19,30], accumulation of some of these TFs could induce genes on a second hierarchical tier. Detectable accumulation of the corresponding transcripts may, however, require longer time periods than analyzed in this or previous studies [30].

Interestingly, our combined analyses of the light-inducible transcriptome and the WCC cistrome revealed 13 genes (S5 Table) that were not light-inducible despite the presence of highly confident WCC binding sites with tandem GATC motifs in their promoters. To support expression of these genes the WCC may cooperate with unknown TFs, which were not active under the experimental conditions analyzed.

A bipartite sequence motif is highly enriched in binding sites of SUB1 (Fig. 2F, upper panel). The first half of this motif (a/cGATc/g) is related to binding motifs of GATA-family proteins. The second part of the motif (a/cTGc/t) is located a full helical turn of the DNA away (center to center) and could reflect an additional sequence-specific contact of SUB1 or correspond to the binding site of an interaction partner of SUB1. We identified FF7 as dynamic interaction partner of SUB1. FF7 contains a putative O-acetyl transferase domain and a Zn2Cys6 binuclear cluster DNA binding domain that binds to AAGCGC motifs and not to a/cTGc/t. An interaction partner other than FF7 was not detected in our affinity purified SUB1 preparation and SUB1 is not predicted to harbor a second DNA binding domain in addition to its GATA-type Zn-finger. Hence, the functional role of the a/cTGc/t remains elusive.

The large fraction of SUB1 binding sites overlapping with FF7 binding sites (∼70%) and the large fraction of SUB1-affected genes co-regulated by FF7 (∼ 60% of the light-inducible and ∼ 47% of non light-inducible genes) suggests that SUB1 might generally cooperate with FF7. However, FF7 has ∼4-fold more genomic binding sites than SUB1. Hence, FF7 seems to have a broader role and it may cooperate with other TFs in addition to SUB1, consistent with the more severe phenotype of Δ*ff7* (reduced conidiation and female fertility) in comparison to a Δ*sub1* strain. When SUB1 and FF7 binding sites are sufficiently close, individually weak interactions of SUB1 and FF7 with their cognate binding sites as well as weak interactions of SUB1 and FF7 (as detected by pull-downs) might be mutually stabilized to specify the subset of genes regulated by these two TFs.

Pathways and cues regulating SUB1 activity are not known. The expression levels of SUB1-affected light-inducible target genes were roughly proportional to SUB1 abundance, suggesting that WCC and SUB1 act synergistically. At light-inducible genes that are independent of SUB1 the corresponding activity might not be rate limiting or provided by other, unidentified TFs with or without the help of FF7. Combinatorial cooperation of TFs with the WCC would allow differential regulation of subsets of the large group of light-inducible genes. Thus, WCC could regulate the fold induction of genes, i.e. light versus dark ratio, while cooperating TFs such as SUB1 might synergistically support gene expression in light and in dark. Hence, the apparent set of genes that respond in significant manner to light may crucially depend on the activity of TFs cooperating with the WCC.

## Materials and Methods

### *Neurospora* strains

*Neurospora* strains; *wt* (FGSC #2489), Δ*sub1* (FGSC #11127), Δ*wc2* (FGSC #11124), Δ*ff7* (FGSC #11073), *wc1* ^*mut*^ (FGSC #4398), *bd* (FGSC #1859), *his-3* (FGSC #6103) used in this study were acquired from FGSC. *wc1* ^*+*^ (FGSC #9718) was used to generate *ff7*_*FlagHis*_, *sub1*_*FlagHis*_ and *wc1* ^*+*^ *qa2*::*sub1*_*Flag*_ strains. *wc1* ^*mut*^ was used to generate *wc1* ^*mut*^ *qa2*::*sub1*_*Flag*_. *bd* Δ*wcc*, *his-3* [52] was used for integration of *ccg1*::*sub1* into the *his-3* locus to obtain *bd* Δ*WCC ccg1*::*sub1*. *his-3* strain was crossed to Δ*sub1* to create Δ*sub1*,*his-3* strain that was used to generate *tub*::*sub1* and *ccg1*::*sub1*strains. Δ*sub1*,*his-3* strain transformed with empty vector was used as a *sub1* KO strain in RNA measurements together with *ccg1*::*sub1* and *tub*::*sub1* strains.

### Culture conditions

Standard growth medium contained 2% glucose, 0.5% L-arginine, 1× Vogel's medium, and 10 ng / mL biotin. For light-induction experiments, indicated strains were grown in petri plates until mycelial mats formed. Mycelial pads (1 cm) were cut out and grown for 1 day in light at 25°C and transferred to darkness for 24 h before cultures were exposed to light (100 μE) and harvested after the indicated time periods. For replicate 1 of the light-induction experiment *wt*, Δ*sub1* and Δ*ff7* strains were analyzed whereas *wt* and *ccg1*:*sub1* strains were analyzed for the replicate 2. For the QA induction experiments 0.3% QA (final) was added to 24 h dark grown and constant light grown cultures. Samples were harvested before and 4 hours after the addition of QA. For nucleosome mapping conidia of *wt*, Δ*sub1*, Δ*wc2* and Δ*ff7* (strains were inoculated in 200 ml media, grown in light for 2 days and transferred to darkness for 24 h. Dark grown and 20 min light exposed cultures were crosslinked with 0.5% paraformaldehyde (FA) for 10 min. FA was quenched with 125 mM glycine for 5 min. Δ*ff7* was not included into the replicate 2 nucleosome mapping.

### Generation of knock-in cassettes and *Neurospora* transformations

The yeast in vivo recombination system [53] was used to generate *sub1*_*FlagHis*_, *ff7*_*FlagHis*_ *and qa2*::*sub1*_*Flag*_ *strains*. Transformation of *Neurospora* was performed as described [53]. Primers are listed in S6 Table.

### Protein analysis

Extraction of proteins and subcellular fractionation were performed as described [54]. SUB1 rabbit antibody was generated against the peptide “RKRQLEQRSIRPKPTDDRN”. H2A antibody was generated against the peptide “CHQNLLPKKTGKTGKNASQEL” Western blotting was performed as described [55]. Protein concentration was estimated by measuring absorption at 280 nm (NanoDrop, PeqLab). Enhanced chemiluminescence signals were detected with X-ray films (Fuji Film Tokyo, Japan).

### RNA analysis

RNA was prepared with peqGOLD TriFAST (peqLab, Erlangen, Germany) and reverse transcribed with the QuantiTect Reverse Transcription Kit (QIAGEN, Hilden, Germany). Transcript levels were analyzed by quantitative real-time PCR in 96-well plates with the StepOnePlus Real-Time PCR System (Applied Biosystems). TaqMan Gene Expression Master Mix (Applied Biosystems) and UPL probes (Roche) were used. Primers and probes are listed in S6 Table.

### MNase digestion for nucleosome mapping

The previously published MNase digestion protocols [56,57] were optimized for *Neurospora*. 400 mg ground mycelial powder from each culture was resuspended in 3.75 ml MNase digestion buffer (250 mM sucrose, 60 mM KCl, 15 mM NaCl, 15 mM Tris-HCl pH 7.4, 3 mM MgCl_2,_ 1 mM CaCl_2,_ 0.2% NP-40) with freshly added 0.5 mM DTT, 0.5 mM Spermidine and protease inhibitors (EDTA-free, Roche). The suspension was mixed by vortexing and incubated on ice for 5 minutes. Next 750 μl aliquots were distributed to five 1.5 ml Eppendorf tubes. MNase powder (Sigma-N3755-500) was resuspended in 850 μl MNase resuspension solution (10 mM HEPES-KOH pH 7.6, 50 mM KCl, 1.5 mM MgCl_2_, 0.5 mM EGTA, 10% glycerol). Aliquots were digested with different amounts (0 [control], 0.75, 1.5, 3, 6U) of MNase to cover both, sensitive and resistant sites. All samples were incubated at 25°C for 1 hour with shaking at 400 rpm in a Themo-mixer. The reaction was stopped by adding stop buffer (final 0.2% SDS, 10 mM EDTA pH 8.0). Samples are centrifuged at 20000 g for 20 min to pellet the cell debris. Supernatants were transferred to new tubes and 2 μl RNAse Cocktail Enzyme mix (Life Technologies, AM2286) was added and incubated at 37°C for 45 minutes to degrade RNA. To degrade proteins, 15 μl proteinase K solution (Life Techonolgies, AM2548) was added and samples were incubated at 65°C for 2 hours. DNA was precipitated with EtOH in the presence of 40 μg glycogen (Thermo) and further cleaned by using PCR clean-up kit (Promega). Aliquots were analyzed by electrophoresis in a 1.7% Agarose gel (80 volts for 1 hour) to visualize nucleosomal DNA ladders. Paired-end libraries for sequencing were prepared as described below.

### MNase-WC2-ChIP

Neurospora cultures were crosslinked with 1% FA for 10 min. FA was quenched 5 min with 125 mM glycine. ∼ 400 μl ground mycelial powder was resuspended and digested in 600 μl MNase digestion buffer for 1 h at 25°C with 15 U of MNase (Sigma-N3755-500) (In addition an independent sample from a 1 min light-induced culture was digested with 5 U MNase). The reactions were stopped by adding final 5 mM EDTA pH 8.0. Then 400 μl ChIP lysis buffer (50 mM HEPES, 150 mM NaCl, 1 mM EDTA, 1% Triton-X, 0.1% SDS and 0.1% NaDOC) was added and samples were centrifuged at 4°C, 15000 g. The rest of the ChIP protocol was performed as described [19]. DNA for ChIP-seq was pooled from two-independent experiments.

### Tandem chromatin immunoprecipitation with FLAG-His and protein A/Calmodulin

Light-induction was performed for the indicated time periods and cultures were cross-linked in constant light with 1% FA for 15 min. FA was quenched with 125 mM glycine for 5 min. 6 aliquots of 600 μl ground mycelial powder were used for each time point for TAP-WC2-ChIP-seq. Mycelia were dissolved in 1 ml ChIP lysis buffer (50 mM HEPES pH 7.4, 150 mM NaCl, 1 mM EDTA, 1% Triton-X, 0.1% SDS and 0.1% NaDOC) with freshly added 0.5 mM DTT and protease inhibitors (Roche cOmplete Protease Inhibitor Cocktail-EDTA free). Sonification was performed with SonoLab^TM^ Covaris Version 7.0.20.0 (average incident power 36 watt, peak incident power 180 watt, duty factor 20 percent, cycles / burst 200 count and duration 160 seconds). Samples were centrifuged at 15000 g for 20 min at 4°C. Supernatants of the six aliquots per time points were combined and incubated with 100 μl IgG Sepherose/time point (GE Healthcare) for 3 h at 4°C. Beads were washed 2x with ChIP lysis buffer, 1x Lindet (250 mM LiCl, 1% NP-40, 1% NaDOC, 1 mM EDTA, 10 mM Tris / HCl; pH 8) and 2x with TAP buffer (50 mM Tris–HCl, pH 7.5, 150 mM NaCl, 1.5 mM MgCl2, 0.1% NP-40, 0.5 mM DTT). Elution was performed with two consecutive digestions with 50 U TEV protease (Invitrogen) at 16 C° 2 h and ON at 4 C°. Final 3 mM CaCl_2_ was added to the combined elution and incubated with 130 μl Calmodulin Sepharose 4B (GE Healthcare) for 3.5 h. Beads were washed 5 times with TAP buffer with 3 mM CaCl_2_. The elution and DNA extraction was performed as described [19]. DNA for ChIP-seq was collected from three-independent experiments.

Tandem ChIP with Ni-NTA enrichment, followed by anti-Flag immunoprecipitation was performed as described [33]. Primers and probes used for the ChIP-PCR are listed in S6 Table.

### RNA sequencing and data analysis

NEBNext Ultra RNA Prep kit with NEBNext Multiplex oligos was used for cDNA preparation at Bioquant Deep Sequencing Core Facility. PolyA selection was performed at the beginning of the protocol. The size and the quality of the libraries were checked with a 2100 Bioanalyzer. Un-paired sequencing with 50 bp read length was performed with a HiSeq 2000 at GeneCore EMBL Heidelberg. Raw reads can be accessed at SRA database under the accession numbers listed in S7 Table.

50 bp long raw reads were mapped to *Neurospora crassa* genome NC10 by using Bowtie [58]. Maximum 3 mismatches were allowed for the mapping. Reads mapping to more than one site were discarded. Gene expression was quantified by the number of reads falling into annotated exons. Normalization between samples was performed by using the size factor formula as described [59]. Genes with low RNA levels (lower 20% of all annotated genes) were excluded from further analysis.

In order to identify significant light-induced genes, differential gene expression analysis was performed. Read counts of the genes were assumed to follow negative binomial (NB) distribution, Gi≈NB(μi, σi), where μi is mean and σi is variance. Since no replicates information were available, σi was estimated based on the mean and variance correlation. A “locfit” R package was used to fit the relationship between the mean and variance. By adapting the Robinson and Smyth Exact Test [60], the two side p-value can be computed as follow,

$$p=\frac{\sum_{f\left(a,b\right)\leq f\left(G_{treat},G_{control}\right)}f\left(a,b\right)}{\sum f\left(a.b\right)}$$

where $a+b=G_{treat}^{i}+G_{control}^{i}$, $a,b\in 0..\left(G_{treat}^{i}+G_{control}^{i}\right)$. $G_{treat}^{i}$ is the read counts of *i*_*th*_ gene in treatment condition, $G_{control}^{i}$ is the read counts of *i*_*th*_ gene in control condition, *f*(*a*,*b*) is the product of *f*(*a*) and *f*(*b*), which can be computed using dnbinom of R package. After finding the genes that have a significant (p < 0.05) increase in the reads in either 30, 60 or 120 minutes compared to DD reads, we set another cut off (2 fold) to further filter the candidates. In order to identify genes that show impaired light-induced in *Δsub1* light-induced time points were compared and p values were calculated as described above. The time points that have significantly lower reads (p < 0.05) in the knock-out strains were identified. Similar analysis and p values were used to identify up-regulated genes in *ccg1*:*sub1* compared to *wt* (replicate 2).

We analyzed ChIP-seq data from Cesbron et al. to assess the kinetics of RNAPII recruitment to 519 light-inducible genes (RNA-seq replicate 1). Significantly increased levels (p < 0.05) of transcribing RNAP (RNAPII-S2P) were detected at 221 genes already 1, 5 and 10 min after a 1 min light-pulse.

### Nucleosome mapping and data analysis

Libraries from purified MNase-digested DNA were prepared without size selection to detect nucleosomal DNA and putative footprints of transcription factors. NEBNext Ultra™ DNA Library Prep Kit for Illumina (E7370L) was used for library preparation at the Bioquant Deep Sequencing Core Facility by using manufacturer`s instructions. The size and the quality of the libraries were analyzed with a 2100 Bioanalyzer. Paired-end sequencing with 100 bp read length was performed with a HiSeq 2000 at BGI, Hong Kong. Raw reads can be accessed at SRA database under the accession numbers listed in S7 Table.

For nucleosome mapping the fragment length was set to be between 100 bp and 1000 bp with forward and reverse conformation. For mismatches and multiple alignments the same settings were used as in the single end mapping. The non-mapped reads were further processed to remove the adapters, mapped to the genome again and analyzed independently. The middle 50 bp of the pair end position was used to generate the wiggle file format genome nucleosome coverage. Wig files were visualized with IGV genome browser [61]. The normalization factor was computed by using 90^th^ percentile of each experiment. Smoothing was carried out by using Kernel Regression Smoother package of R. 1500 bp upstream and downstream of annotated TSS position were used for genome wide nucleosome coverage analysis. The center of the +1 nucleosome of genes was defined by the maximum read coverage in a window of 200 bp around the annotated TSS. Each nucleosome was estimated to cover 176 bp, and the nucleosome coverage was estimated by the area under the curve.

To analyze the nucleosome coverage at the transcription factor binding sites (BS) the nucleosome mean coverage at the summit of the BS was calculated. As the position of a BS relative to a TSS follows a NB, a random NB using the same parameter was generated. The background nucleosome mean coverage was then computed by using the random relative position of BS to TSS. The nucleosome mean coverage was determined by using the normalized nucleosome mean coverage and plotted as ratio to remove the possible bias related to the promoters.

### ChIP-Sequencing and data analysis

ChIP DNA libraries were prepared with NEBNext ChIP-Seq Library Prep Reagent Set for Illumina with NEBNext Multiplex oligos at Bioquant Deep Sequencing Core Facility. A 2100 Bioanalyzer was used to check the quality and size of the libraries. Un-paired sequencing with 50 bp read length was performed with a HiSeq 2000 at BGI, Hong Kong.

Mapping was performed as in RNA-seq analysis. In order to identify peaks a sliding window of 150 bp with a step of 50 bp was used to scan the genome and quantify the read intensity. The read intensity was normalized by using the total mapped reads. Wig files were visualized with IGV genome browser [61]. The significantly enriched windows were computed by fitting the read intensity to a Poisson distribution. A ChIP binding site was called if 4 continuous windows were statistically higher (p value < 1e-05) compared to the control ChIP-seq. FGSC #9718 strain was used as a background to identify the significant windows for FLAG-HIS ChIP-seq. Dark ChIP of each experiment was used as a background for TAP-WC2 and MNase-WC2 ChIP. Another cut off was determined based on the coverage of the identified ChIP peak. We excluded the binding sites with low enrichment (< 1.5 fold, < 90^th^ percentile of the coverage) by comparing the peak coverage to general coverage of the ChIP-seq. Raw reads can be accessed at SRA database under the accession numbers listed in S7 Table.

Upstream and downstream genes were analyzed for the annotation of the peaks. The gene was annotated to a peak if there was a peak detected within 1000 bp upstream of the TSS and 500 bp downstream of TSS. If the peak was not close to a promoter of any gene, the binding site was annotated to the nearest genes with the expected orientation (i.e. binding sites are upstream of the annotated TSS) with no distance limitation. If there were no genes in the upstream/downstream of the peak with the correct orientation, the peak was not annotated to any gene.

The SUB1 binding sites of DD and 30 min light-induction samples were merged based on the coordinate location to find light-induced SUB1 binding sites. Using linear regression between the DD and 30 min light-induction read intensity of the peaks, light-regulated SUB1 binding sites were identified. The linear regression was fitted using R, and the clustering was based on the 80% confidence prediction interval.

Motif analysis was done by using MEME motif search software [35]. 300 bp DNA regions surrounding the summit of the ChIP-seq peaks were used. For the WCC ChIP-seq “GATC” motif the pair wise distribution was calculated within these 300 bp regions.

## Supporting Information

S1 Fig A. Western-blot showing rhythmic expression of SUB1 in *wt* during a two-day circadian time course in darkness. **B**. Western blot showing light-induced SUB1 levels in *wt* and *Δwc2* strains. **C**. Heat-map of RNA-seq analysis of all light-inducible genes in *wt* (n = 519). **D**. Heat-maps of 593 non-light inducible genes with lower average RNA levels in *Δsub1* compared to *wt*. **E**. Light-induced expression of *vvd* RNA is not affected by SUB1. Quantification of *vvd* RNA levels by RT-PCR (± SEM, n = 5) in *wt* (black line) and *Δsub1* (red line) strains at the indicated time periods after light-exposure of mycelial cultures. 28s rRNA was used for normalization. The *vvd* RNA level of *wt* at t = 0 min (dark) was set to 1. **F**. Light induced recruitment of initiating RNA polymerase II (RNAPII) to *rds1* and *hyr1* in *wt* and *Δsub1* strains. ChIP was performed with antibodies recognizing RNAPII phosphorylated at serine-5 of the C-terminal heptad repeats (RNAPII-S5P). 28s rDNA was used for normalization. Graphs show average of two independent experiments. **G**. Western blot showing SUB1 levels of the *wt* cultures analyzed in F. **H**. Box-plots showing the RNAPII-S2P occupancy (elongating RNAPII phosphorylated at serine-2 of the C-terminal heptad repeats) of SUB1-dependent (left panel) and SUB1-independent (right panel) light-inducible genes at the indicated time points (in minutes) after a 1 min light pulse. The ChIP-seq data are from Cesbron et al 2015. See methods for details of the analysis.
(PDF)

S2 Fig A-B. Genome-wide analysis of WCC binding sites. Heat-maps showing the light-induced WC2 occupancy at binding sites identified by **(A)** TAP-WC2 ChIP-seq and **(B)** MNase-WC2 ChIP-seq. For MNase-WC2 ChIP, samples were treated with 15 U MNase (or 5 U MNase when indicated) prior to immunoprecipitation with WC2 antibody. **C**. WCC binding motif identified by MEME. The motif is found in 71 out of 92 sites. **D**. Many true WCC binding site are not detected by WC2 ChIP-seq. Left panels: Wig files of WC2 occupancy at light-inducible promoters with potential WCC binding sites (red boxes) that were not detected by the peak-calling algorithm of the WC2 ChIP-seq analysis. Right panels: WC2 ChIP-PCR analysis showing light-induced binding of WCC to these sites. The location of GATC motifs is shown at the bottom of each panel. **E**. Heat-maps showing light-induced binding of SUB1 to a subset of sites (n = 50). **F**. Heat-maps showing SUB1_FLAGHIS_ ChIP-seq signals at the 92 highly confident WCC binding sites. **G-I**. ChIP-seq coverage of **(G)** SUB1 and **(I)** WCC at promoters of light-inducible genes. Dotted lines show SUB1-independent light-inducible genes (n = 330) whereas solid lines show SUB1-dependent light-inducible genes (n = 189).
(PDF)

S3 Fig A. Nucleosome occupancy at binding sites of WCC (n = 92) (upper panels) and SUB1 (n = 617) (lower panels) in *wt* (red), *Δsub1* (green) and *Δwc2* (blue) in dark (left panels) and 20 min after light-exposure (right panels). Data shown in Fig. 3A and C are re-plotted for comparison of samples at same time point. **B**. Nucleosome occupancy in replicate 2 at binding sites of WCC (n = 92) and in *wt* (red), *Δsub1* (green) and *Δwc2* (blue) strains in dark (dotted lines) and 20 min after light-exposure (solid lines). **C**. Sub-nucleosomal footprint at binding sites of the WCC (left panel) and SUB1 (right panel) in dark (dotted lines) and 20 min after light-exposure (solid lines) of cultures from replicate 2. Sequence coverage of small (< 100 bp) MNase-resistant fragments in *wt* was normalized to mean background coverage.
(PDF)

S4 Fig A. Wig files showing the nucleosome position and occupancy at the *hyr1* locus (left panel) and the *vvd* locus (right panel) in *wt*, Δ*sub1* and Δ*wc2* strains in the dark and after light-exposure. The MNase-WC2 ChIP-seq signal is shown below the nucleosome signals. Numbers on the ChIP-seq panels indicate the maximum coverage shown in the wig file. **B**. Wig files showing the zoom-out versions of the regions shown Fig. 4D with light-induced nucleosome eviction at WCC binding sites that are not associated with transcription of a nearby gene. RNA-seq reads in dark and 30 min after light exposure mapped to these regions are shown in the bottom panels. The TAP-WC2 ChIP-seq (blue) signal is shown in the top panels.
(PDF)

S5 Fig A. Western blot showing SUB1 levels in *wc1* ^*+*^ and *wc1* ^*mut*^ strains. **B**. Quantification of *hyr1* RNA levels by RT-PCR in *wc1* ^*+*^ and *wc1* ^*mut*^. Expression levels were normalized to *tubulin* RNA (± SEM, n = 4). The RNA level of dark grown *wc1* ^*+*^ (- QA) was set to 1. **C**. ChIP-PCR analysis of FLAG-SUB1 showing binding of SUB1 to the *rds1* promoter in *wc1* ^*+*^ *qa2*::*sub1*_*FLAG*_, *wc1* ^*mut*^ *qa2*::*sub1*_*FLAG*_ and *Δsub1* strains. Two-step ChIP was performed with FLAG and subsequently SUB1 antibodies. 28s rDNA was used for normalization. The maximal signal (*wc1* ^*+*^ *qa2*::*sub1*_*FLAG*_ in light, + QA) was set to 1 (± SEM, n = 3). **D-E**. RT-PCR measurements of kinetics of light-induced accumulation of **(D)** *rds1* RNA and **(E)** *NCU00309* RNA in *wt*, *Δsub1*, *tub*::*sub1* and *ccg1*::*sub1* strains. 28s rRNA was used for normalization. RNA levels of *wt* at t = 0 min (dark) were set to 1 (± SEM, n = 4). **F**. Light-independent overexpression of SUB1 under control of the *ccg1* promoter. Western blot showing SUB1 levels after light-exposure in *bd* and *bd ΔWCC ccg1*::*sub1* strains. **G-H**. RT-PCR measurement of kinetics of light-induced accumulation of **(G)** *rds1* RNA and **(H)** *NCU00309* RNA in *bd* and *bd ΔWCC ccg1*::*sub1* strains. 28s rRNA was used for normalization. RNA levels of *bd* at t = 0 min (dark) were set to 1 (± SEM, n = 3). **I**. Heat-map of RNA-seq analysis of all genes (657) that were light-inducible in *wt* replicate 2. **J**. Venn diagram showing the overlap of light-inducted genes identified in two independent RNA-seq replicates (*wt*_*rep1*_ *and wt*_*rep2*_). **K**. Heat-maps showing the 121 light-inducible genes with significantly higher RNA levels in *ccg1*::*sub1* compared to *wt*_*rep2*_. **L**. Venn diagram showing the overlap of light-induced genes with reduced RNA levels in *Δsub1* (compared to *wt*_*rep1*_) and light-induced genes with elevated RNA levels in *ccg1*::*sub1* (compared to *wt*_*rep2*_).
(PDF)

S6 Fig A. Median sequence coverage of the SUB1 (blue), FF7 (green) and WCC (red) ChIP at SUB1 (upper panel), FF7 (middle panel) and WCC (lower panel) binding sites. **B**. Wig files of overlapping binding sites of WCC, SUB1, and FF7 at the promoters of light-inducible genes. Numbers on the ChIP-seq panels shows the maximum coverage shown in the wig file. **C**. Expression heat-maps of genes (n = 192) with attenuated light-inducibility in *Δff7* compared to *wt*_*rep1*_. **D**. Kinetics of light-induced expression levels of *rds1* RNA determined by RT-PCR in *wt* and *Δff7* strains (± SEM, n = 4). 28s rRNA was used for normalization. RNA levels of *wt* at t = 0 min (dark) were set to 1. **E**. Heat-maps showing the expression of 278 non-light inducible genes with reduced RNA levels in *Δff7* and *Δsub1* compared to *wt*_*rep1*_.
(PDF)

S1 Table RNA-seq reads of *wt*, *Δsub1* and *Δff7*. (XLSX)

S2 Table WCC and SUB1 ChIP-seq analysis and associated genes. (XLSX)

S3 Table RNA-seq reads of *wt*_*repliate2*_ and *ccg1*::*sub1*. (XLSX)

S4 Table FF7 ChIP-seq analysis and associated genes. (XLSX)

S5 Table WCC peaks with tandem GATC motifs and 28 overlapping binding sites of WCC, SUB1 and FF7. (XLSX)

S6 Table Primers and probes used in the study. (XLSX)

S7 Table SRA accession numbers of each high-throughput sequencing data. (XLSX)
