# Supplementary figures and images for: Combinatorial Control of Light Induced Chromatin Remodeling and Gene Activation in Neurospora
Source: PLoS Genet. 2015 Mar 30;11(3):e1005105. doi: 10.1371/journal.pgen.1005105 (PMC4378982; doi:10.1371/journal.pgen.1005105)

Supplemental Figure 1

**A**

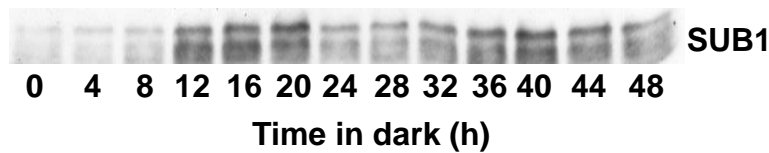

**B**

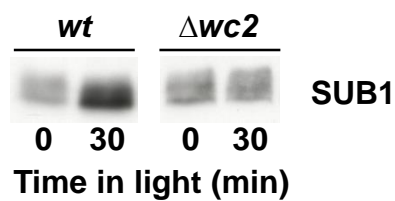

**C**

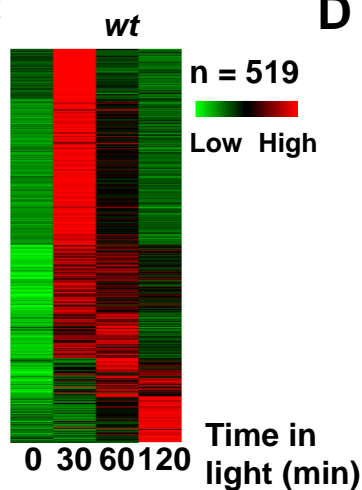

**D**

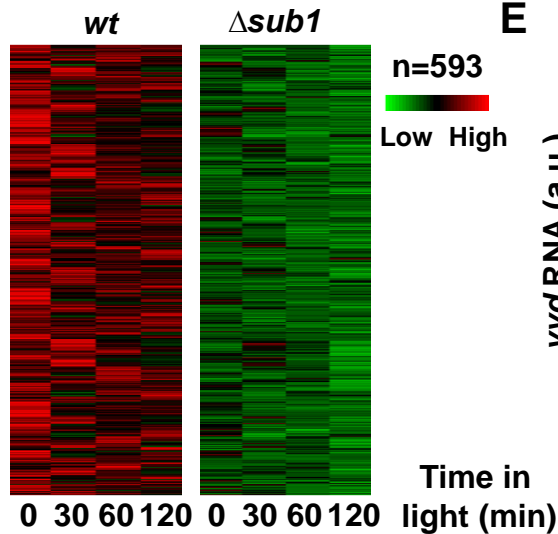

**E**

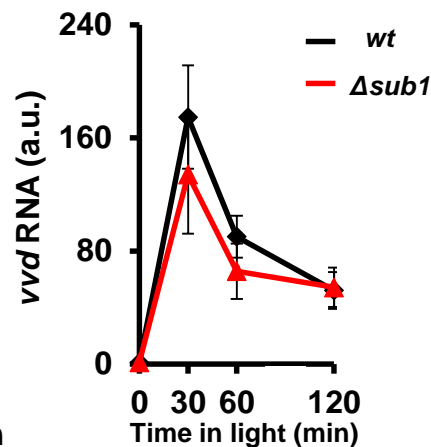

**F**

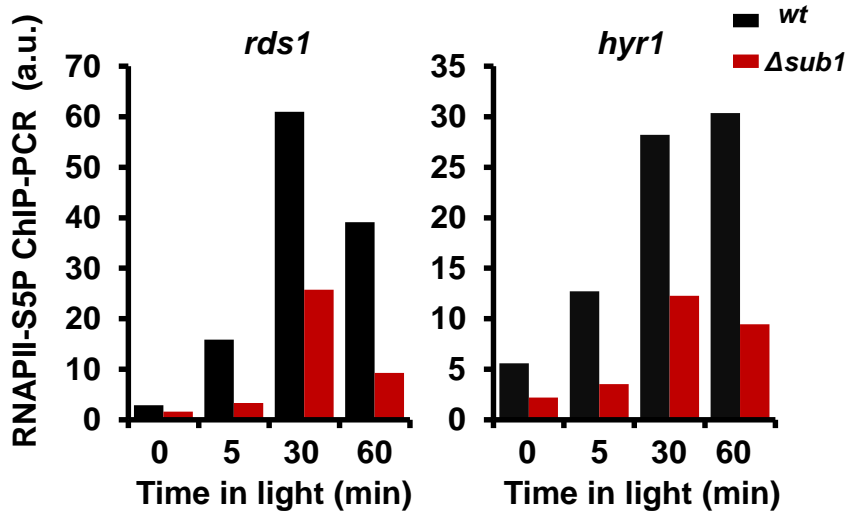

**G**

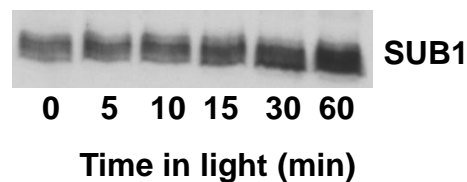

**H**

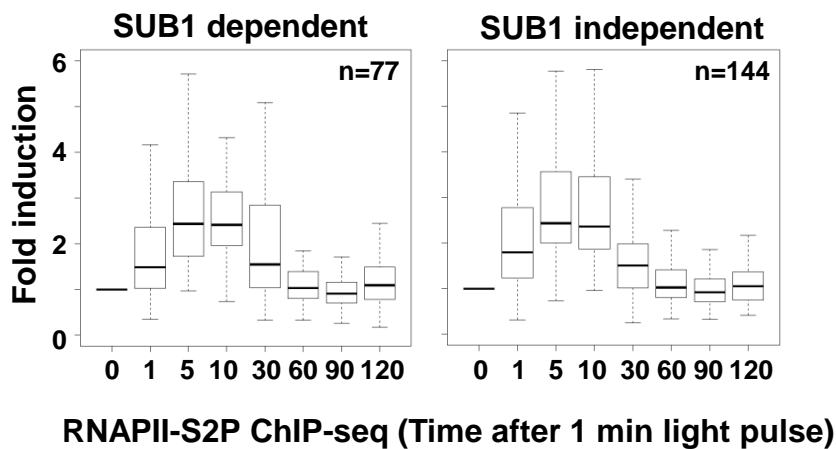

Supplement: S1 Fig — B. Western blot showing light-induced SUB1 levels in wt and Δwc2 strains. C. Heat-map of RNA-seq analysis of all light-inducible genes in wt (n = 519). D. Heat-maps of 593 non-light inducible genes with lower average RNA levels in Δsub1 compared to wt. E. Light-induced expression of vvd RNA is not affected by SUB1. Quantification of vvd RNA levels by RT-PCR (± SEM, n = 5) in wt (black line) and Δsub1 (red line) strains at the indicated time periods after light-exposure of mycelial cultures. 28s rRNA was used for normalization. The vvd RNA level of wt at t = 0 min (dark) was set to 1. F. Light induced recruitment of initiating RNA polymerase II (RNAPII) to rds1 and hyr1 in wt and Δsub1 strains. ChIP was performed with antibodies recognizing RNAPII phosphorylated at serine-5 of the C-terminal heptad repeats (RNAPII-S5P). 28s rDNA was used for normalization. Graphs show average of two independent experiments. G. Western blot showing SUB1 levels of the wt cultures analyzed in F. H. Box-plots showing the RNAPII-S2P occupancy (elongating RNAPII phosphorylated at serine-2 of the C-terminal heptad repeats) of SUB1-dependent (left panel) and SUB1-independent (right panel) light-inducible genes at the indicated time points (in minutes) after a 1 min light pulse. The ChIP-seq data are from Cesbron et al 2015. See methods for details of the analysis. (PDF) [file pgen.1005105.s001.pdf]

Supplemental Figure 3

A

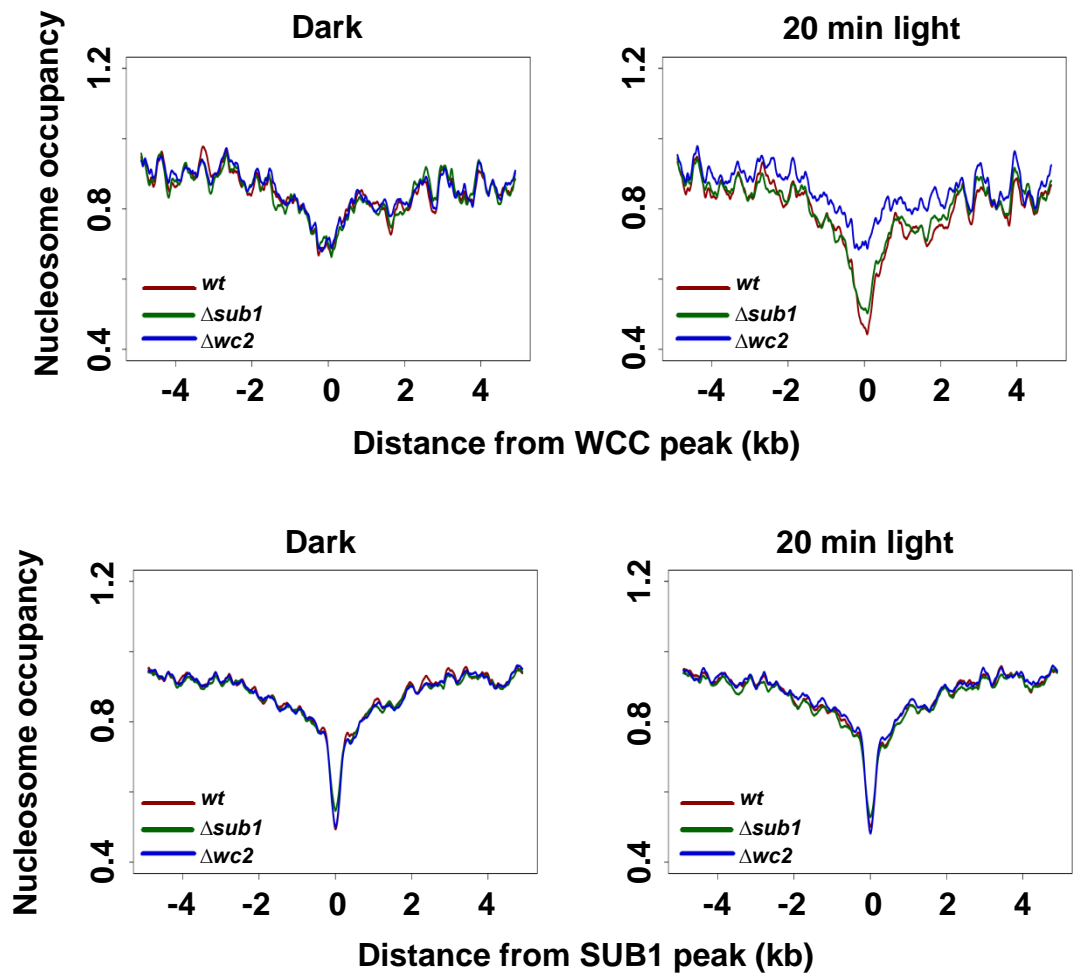

B

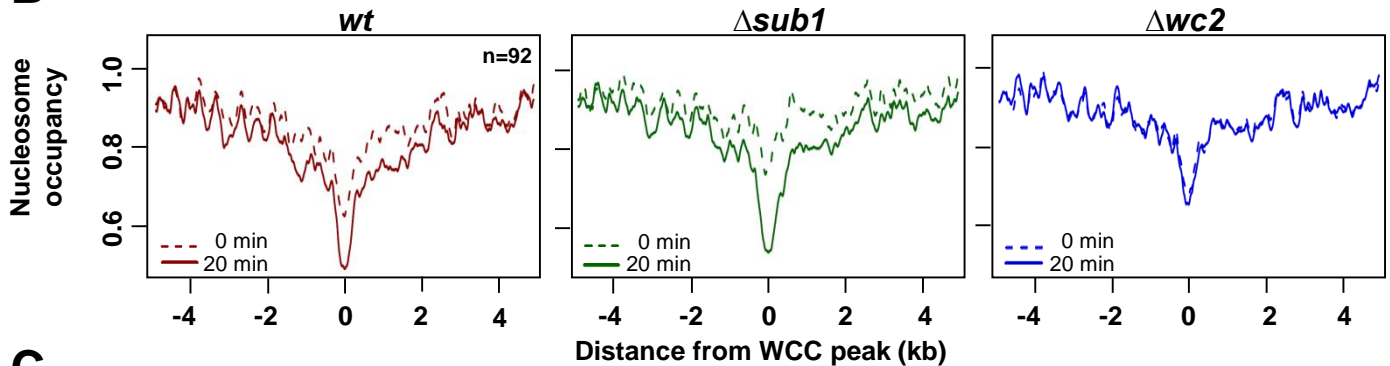

C

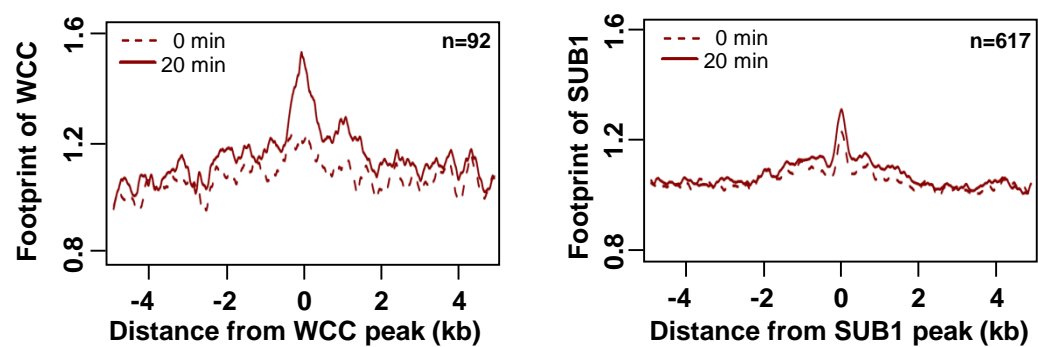

Supplement: S3 Fig — Data shown in Fig. 3A and C are re-plotted for comparison of samples at same time point. B. Nucleosome occupancy in replicate 2 at binding sites of WCC (n = 92) and in wt (red), Δsub1 (green) and Δwc2 (blue) strains in dark (dotted lines) and 20 min after light-exposure (solid lines). C. Sub-nucleosomal footprint at binding sites of the WCC (left panel) and SUB1 (right panel) in dark (dotted lines) and 20 min after light-exposure (solid lines) of cultures from replicate 2. Sequence coverage of small (< 100 bp) MNase-resistant fragments in wt was normalized to mean background coverage. (PDF) [file pgen.1005105.s003.pdf]

Supplemental Figure 4

A

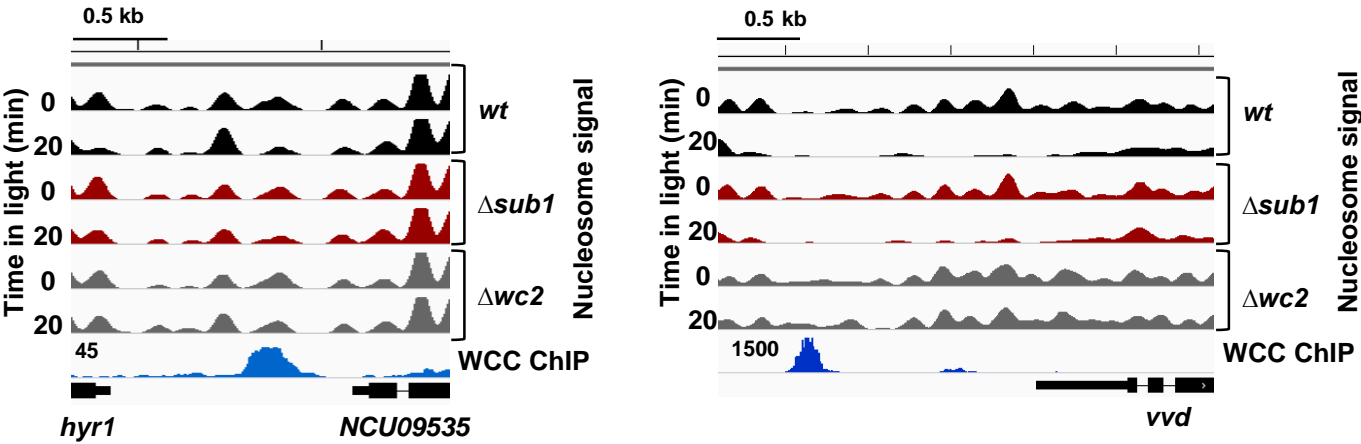

B

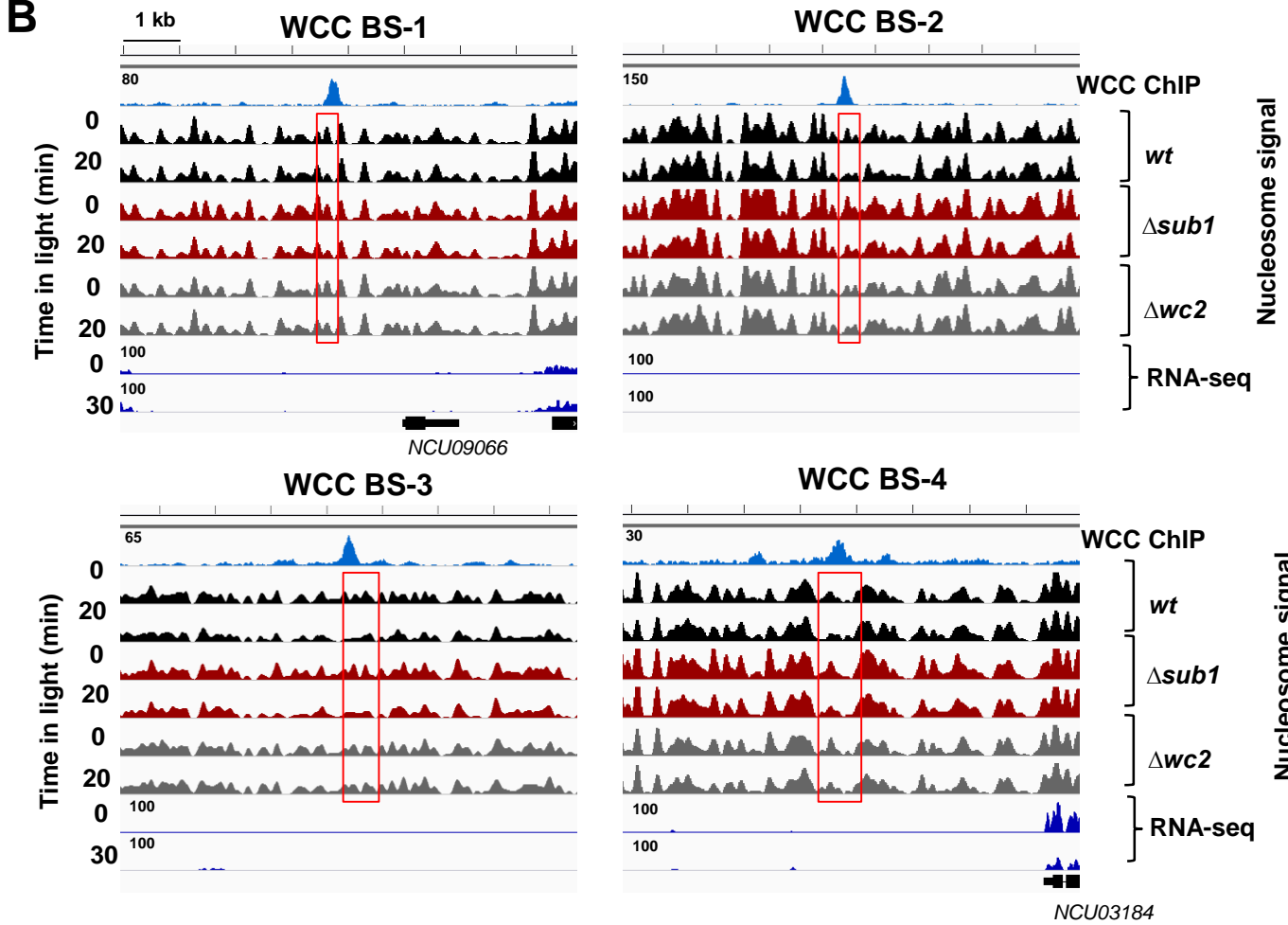

Supplement: S4 Fig — The MNase-WC2 ChIP-seq signal is shown below the nucleosome signals. Numbers on the ChIP-seq panels indicate the maximum coverage shown in the wig file. B. Wig files showing the zoom-out versions of the regions shown Fig. 4D with light-induced nucleosome eviction at WCC binding sites that are not associated with transcription of a nearby gene. RNA-seq reads in dark and 30 min after light exposure mapped to these regions are shown in the bottom panels. The TAP-WC2 ChIP-seq (blue) signal is shown in the top panels. (PDF) [file pgen.1005105.s004.pdf]

Supplemental Figure 5

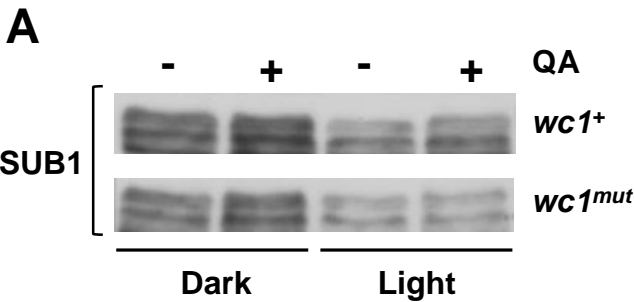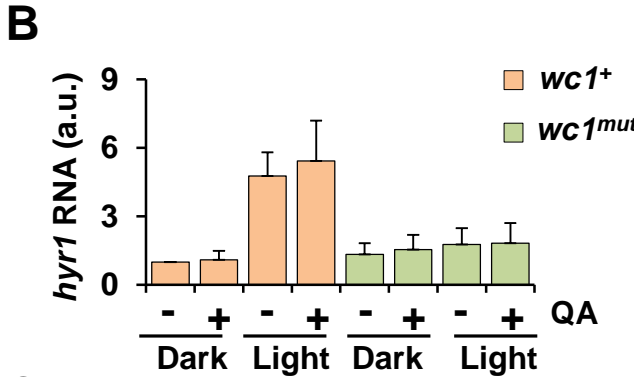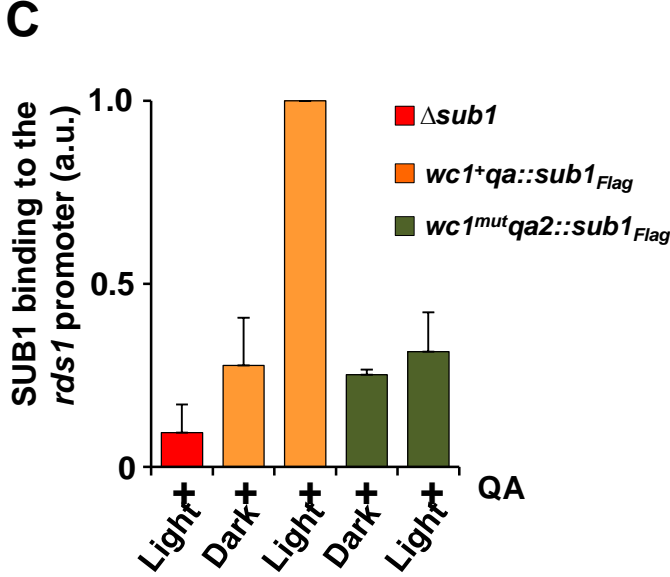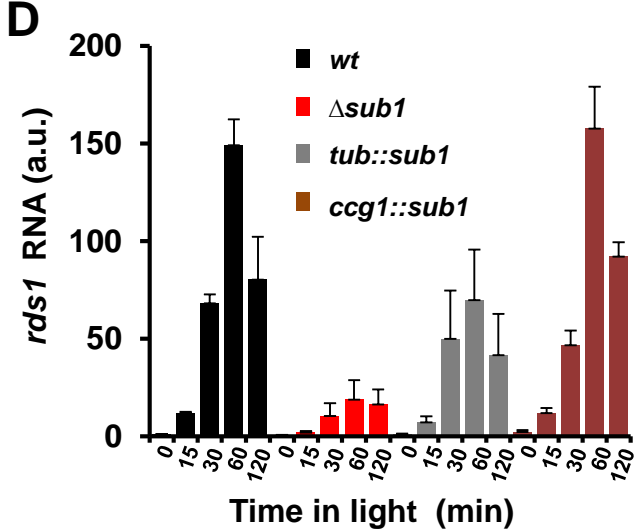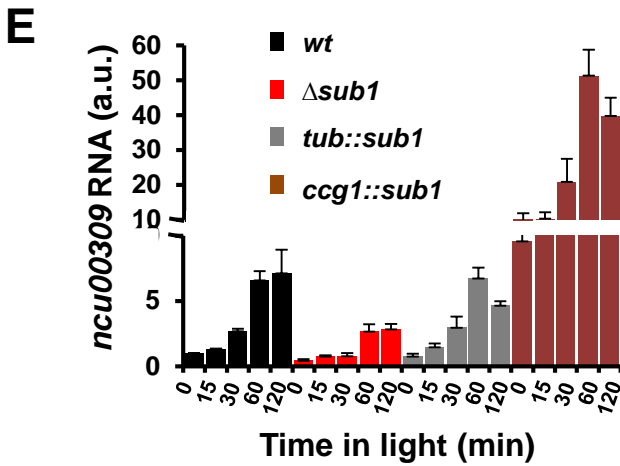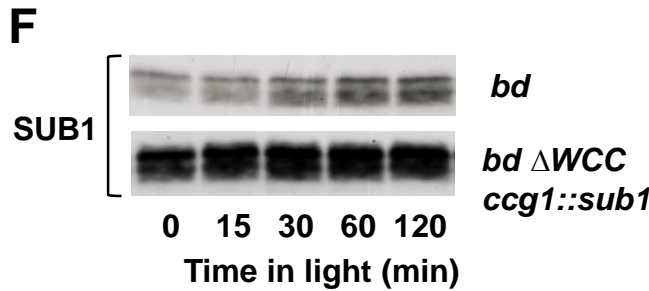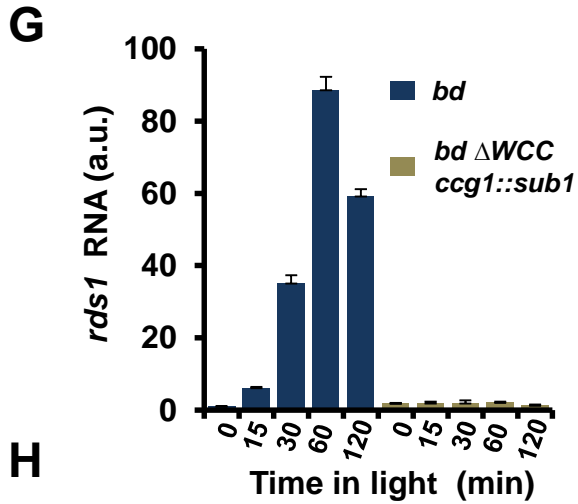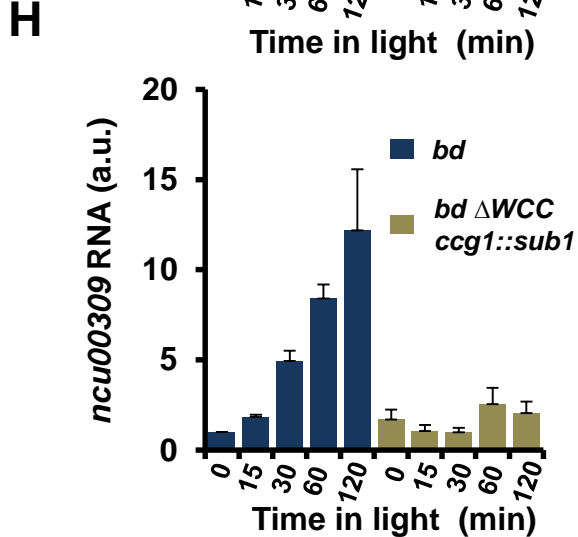

Supplemental Figure 5

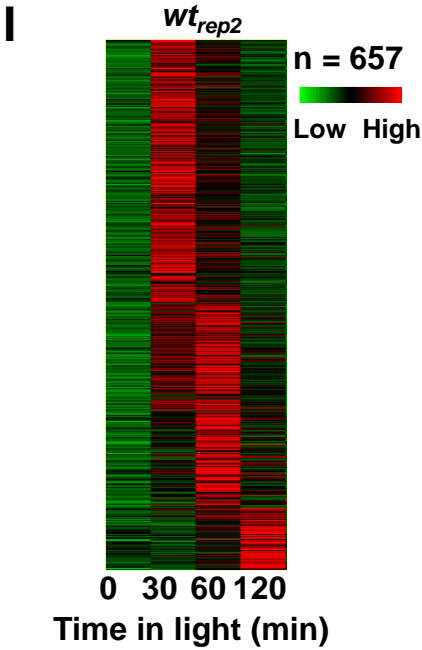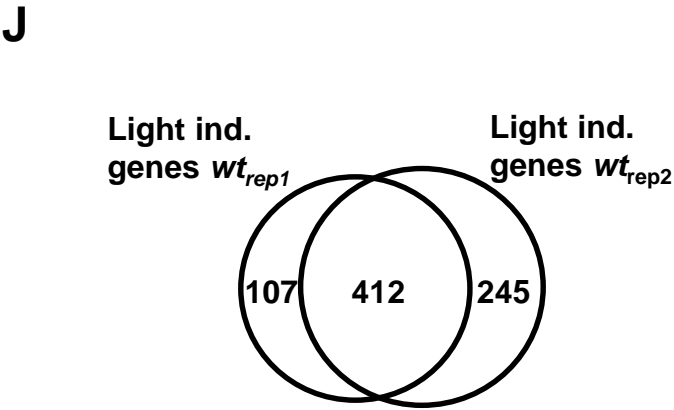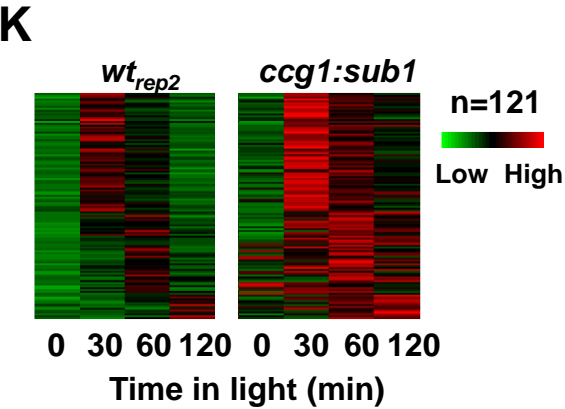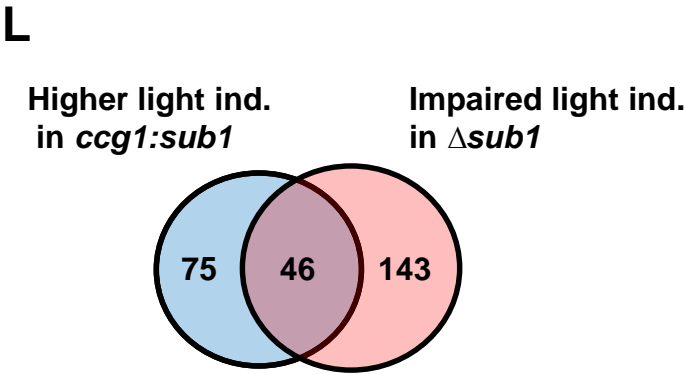

Supplement: S5 Fig — B. Quantification of hyr1 RNA levels by RT-PCR in wc1 + and wc1 mut. Expression levels were normalized to tubulin RNA (± SEM, n = 4). The RNA level of dark grown wc1 + (- QA) was set to 1. C. ChIP-PCR analysis of FLAG-SUB1 showing binding of SUB1 to the rds1 promoter in wc1 + qa2::sub1 FLAG, wc1 mut qa2::sub1 FLAG and Δsub1 strains. Two-step ChIP was performed with FLAG and subsequently SUB1 antibodies. 28s rDNA was used for normalization. The maximal signal (wc1 + qa2::sub1 FLAG in light, + QA) was set to 1 (± SEM, n = 3). D-E. RT-PCR measurements of kinetics of light-induced accumulation of (D) rds1 RNA and (E) NCU00309 RNA in wt, Δsub1, tub::sub1 and ccg1::sub1 strains. 28s rRNA was used for normalization. RNA levels of wt at t = 0 min (dark) were set to 1 (± SEM, n = 4). F. Light-independent overexpression of SUB1 under control of the ccg1 promoter. Western blot showing SUB1 levels after light-exposure in bd and bd ΔWCC ccg1::sub1 strains. G-H. RT-PCR measurement of kinetics of light-induced accumulation of (G) rds1 RNA and (H) NCU00309 RNA in bd and bd ΔWCC ccg1::sub1 strains. 28s rRNA was used for normalization. RNA levels of bd at t = 0 min (dark) were set to 1 (± SEM, n = 3). I. Heat-map of RNA-seq analysis of all genes (657) that were light-inducible in wt replicate 2. J. Venn diagram showing the overlap of light-inducted genes identified in two independent RNA-seq replicates (wt rep1 and wt rep2). K. Heat-maps showing the 121 light-inducible genes with significantly higher RNA levels in ccg1::sub1 compared to wt rep2. L. Venn diagram showing the overlap of light-induced genes with reduced RNA levels in Δsub1 (compared to wt rep1) and light-induced genes with elevated RNA levels in ccg1::sub1 (compared to wt rep2). (PDF) [file pgen.1005105.s005.pdf]

Supplemental Figure 6

A

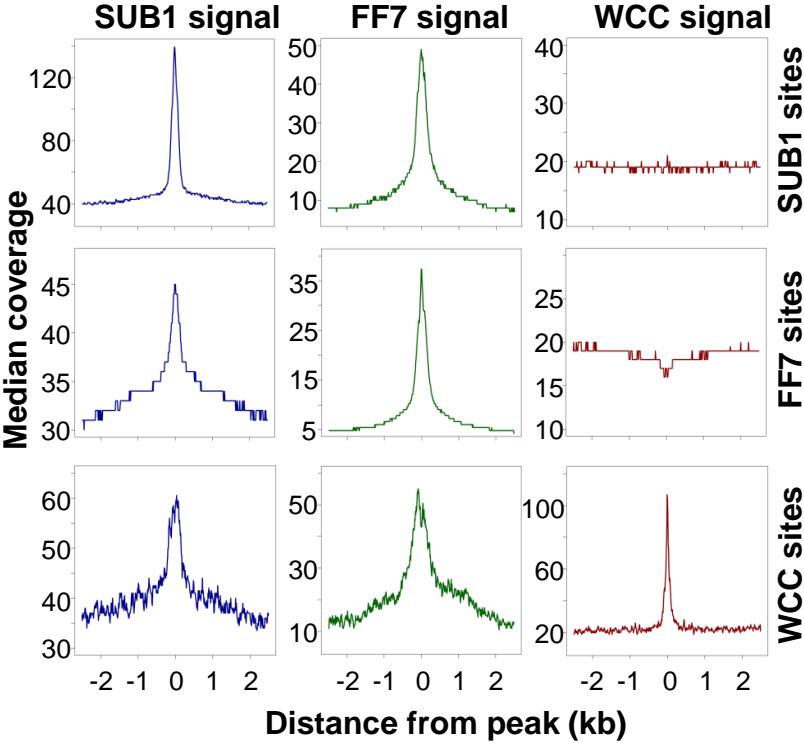

B

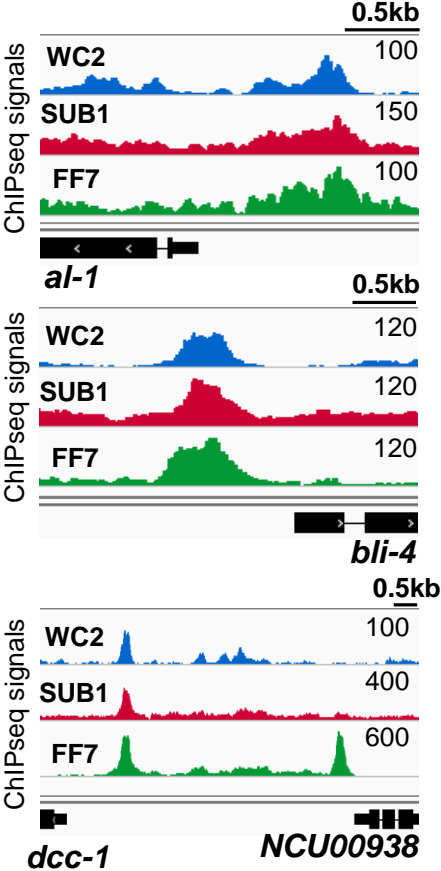

C

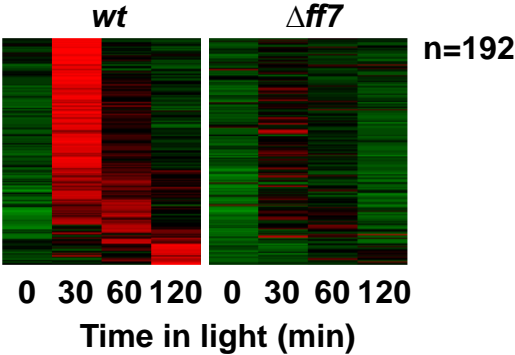

E

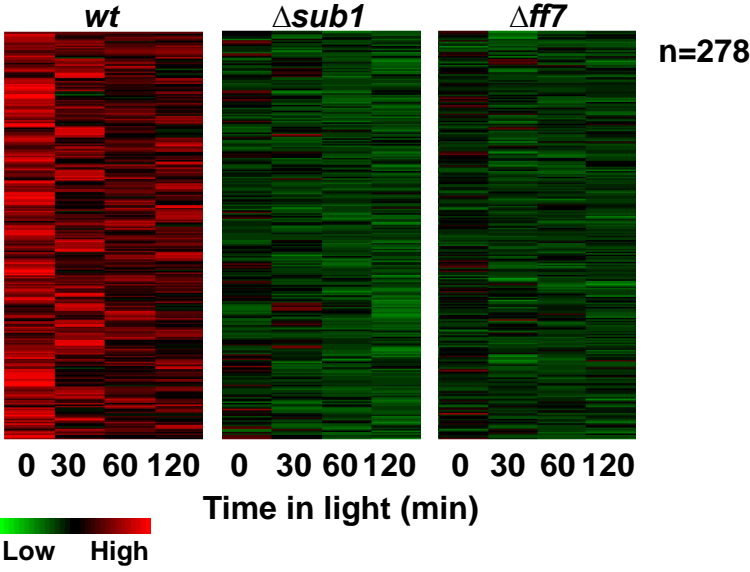

D

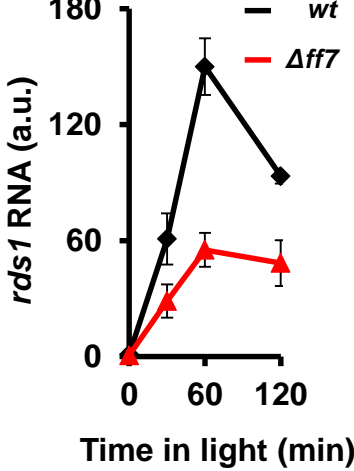

Supplement: S6 Fig — B. Wig files of overlapping binding sites of WCC, SUB1, and FF7 at the promoters of light-inducible genes. Numbers on the ChIP-seq panels shows the maximum coverage shown in the wig file. C. Expression heat-maps of genes (n = 192) with attenuated light-inducibility in Δff7 compared to wt rep1. D. Kinetics of light-induced expression levels of rds1 RNA determined by RT-PCR in wt and Δff7 strains (± SEM, n = 4). 28s rRNA was used for normalization. RNA levels of wt at t = 0 min (dark) were set to 1. E. Heat-maps showing the expression of 278 non-light inducible genes with reduced RNA levels in Δff7 and Δsub1 compared to wt rep1. (PDF) [file pgen.1005105.s006.pdf]
