# Supplementary material for: Combinatorial Control of Light Induced Chromatin Remodeling and Gene Activation in Neurospora
Source: PLoS Genet. 2015 Mar 30;11(3):e1005105. doi: 10.1371/journal.pgen.1005105 (PMC4378982; doi:10.1371/journal.pgen.1005105)

Supplemental Figure 2

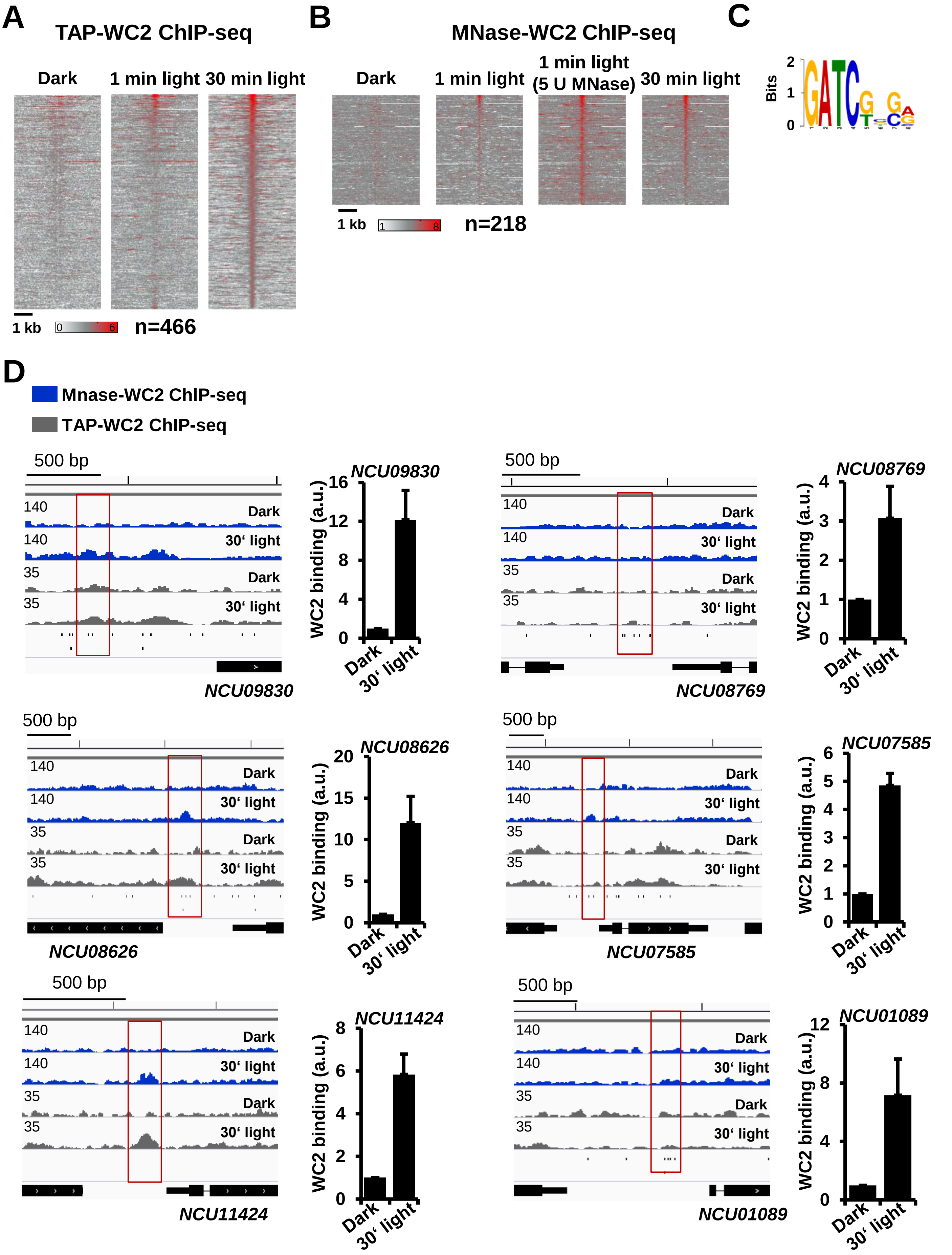

Supplemental Figure 2

E

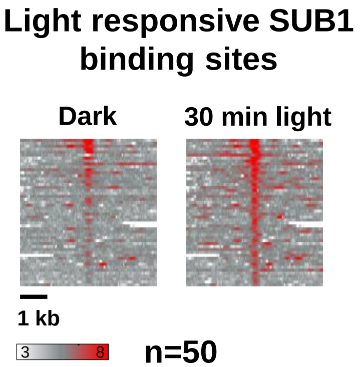

F

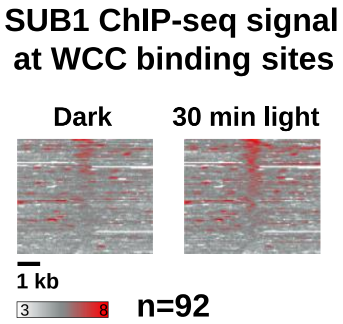

G

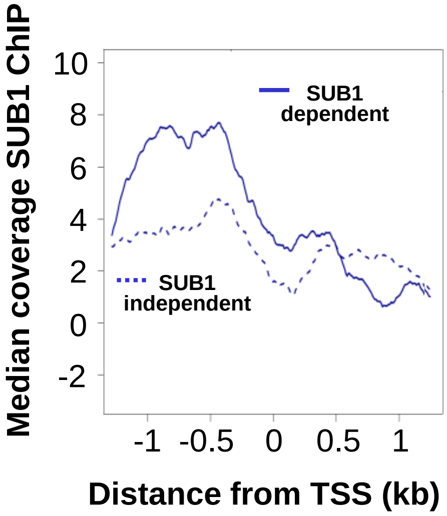

I

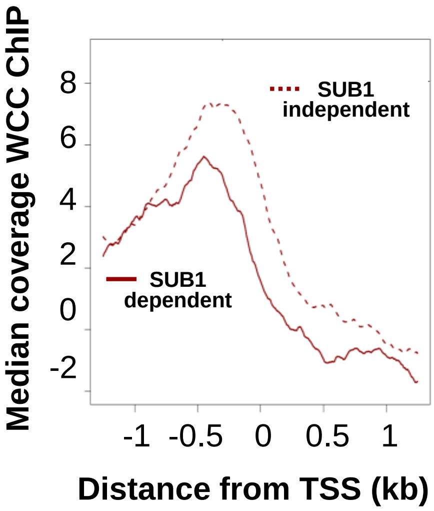

Supplement: S2 Fig — Heat-maps showing the light-induced WC2 occupancy at binding sites identified by (A) TAP-WC2 ChIP-seq and (B) MNase-WC2 ChIP-seq. For MNase-WC2 ChIP, samples were treated with 15 U MNase (or 5 U MNase when indicated) prior to immunoprecipitation with WC2 antibody. C. WCC binding motif identified by MEME. The motif is found in 71 out of 92 sites. D. Many true WCC binding site are not detected by WC2 ChIP-seq. Left panels: Wig files of WC2 occupancy at light-inducible promoters with potential WCC binding sites (red boxes) that were not detected by the peak-calling algorithm of the WC2 ChIP-seq analysis. Right panels: WC2 ChIP-PCR analysis showing light-induced binding of WCC to these sites. The location of GATC motifs is shown at the bottom of each panel. E. Heat-maps showing light-induced binding of SUB1 to a subset of sites (n = 50). F. Heat-maps showing SUB1FLAGHIS ChIP-seq signals at the 92 highly confident WCC binding sites. G-I. ChIP-seq coverage of (G) SUB1 and (I) WCC at promoters of light-inducible genes. Dotted lines show SUB1-independent light-inducible genes (n = 330) whereas solid lines show SUB1-dependent light-inducible genes (n = 189). (PDF) [file pgen.1005105.s002.pdf]
